# A Comprehensive Review on Self-Powered Wearable Photonic Sensors: Materials, Photophysics, and System Integration

**DOI:** 10.3390/s26196277

**Published:** 2026-10-03

**Authors:** Nikolay L. Kazanskiy, Nikita V. Golovastikov, Svetlana N. Khonina

**Affiliations:** 1 Image Processing Systems Institute, NRC “Kurchatov Institute”, 151 Molodogvardeyskaya, Samara 443001, Russia; golovastikov.nv@ssau.ru (N.V.G.); khonina@ipsiras.ru (S.N.K.); 2 Samara National Research University, 34 Moskovskoye Shosse, Samara 443086, Russia

**Keywords:** self-powered photonic sensors, wearable optoelectronics, energy harvesting and autonomy, flexible and stretchable devices, photophysical sensing mechanisms

## Abstract

Self-powered wearable photonic sensors enable energy-autonomous operation by integrating optical sensing, energy harvesting, and signal transduction within a unified platform. These systems exploit photophysical mechanisms such as photovoltaic, photogating, and triboelectric effects to convert ambient or body-derived energy into electrical signals while simultaneously performing sensing. Advances in hybrid semiconductors, two-dimensional materials, and nanocomposites have significantly improved light absorption, carrier transport, and mechanical compliance, enabling integration into flexible and textile-based wearable formats. Device architectures have evolved from simple junctions to heterostructured and tandem configurations that enhance spectral selectivity and energy utilization under variable illumination. However, reliable operation under low-intensity and dynamically changing conditions remains a key challenge, particularly in balancing sensing performance with energy autonomy. This review analyzes the interplay between materials, photophysical mechanisms, device architectures, and system-level design, with emphasis on energy harvesting–sensing co-optimization. Challenges related to mechanical stability, signal reliability, and real-world deployment are discussed, alongside emerging solutions based on adaptive photonic systems and intelligent data processing. These insights provide a framework for next-generation wearable photonic platforms for healthcare, environmental monitoring, and human–machine interfaces.

## 1. Introduction

Wearable photonic sensing technologies have emerged as a cornerstone of next-generation personalized healthcare, environmental monitoring, and human–machine interfacing due to their ability to provide continuous, noninvasive, and high-resolution access to physiological and biochemical signals [1,2,3,4]. Optical sensing modalities such as photoplethysmography [5], spectroscopic analyte detection [6], and fluorescence-based imaging [7] offer intrinsic advantages including high sensitivity, rapid response, and immunity to electromagnetic interference. However, despite substantial advances in device miniaturization, flexible substrates, and integrated optoelectronics, the widespread deployment of wearable photonic systems remains fundamentally constrained by their reliance on conventional battery-powered operation [8,9,10,11]. The need for periodic recharging or replacement introduces limitations in device lifetime, increases form factor and weight, and poses challenges for long-term user compliance and system reliability, particularly in continuous monitoring scenarios.

Battery-dependent architectures also impose significant constraints on system-level design, including rigid packaging, thermal management concerns, and safety considerations associated with electrochemical energy storage in close contact with the human body [12,13]. Furthermore, the mismatch between the intermittent nature of physiological signals and the continuous power draw of sensing and data transmission modules leads to inefficient energy utilization [14]. As wearable systems evolve toward distributed, skin-conformal, and even implantable formats, these limitations become increasingly prohibitive [15]. The challenge is not merely to reduce power consumption, but to fundamentally rethink energy as a central design variable, where sensing, signal processing, and energy management are co-optimized rather than treated as independent subsystems [16].

In this context, energy autonomy has emerged as a critical paradigm for the design of wearable photonic sensors [17,18]. Energy-autonomous systems are those capable of sustaining their operation through energy harvested from the surrounding environment or from the user, thereby eliminating or significantly reducing dependence on external power sources [19]. For wearable photonic platforms, relevant energy sources include ambient light, body heat, mechanical motion, and biochemical processes [20]. Significantly, photonic devices occupy a unique position in this landscape because the same physical mechanisms that enable optical sensing can often be leveraged for energy conversion, as in photovoltaic or photoconductive processes [21]. This dual functionality opens the possibility of intrinsically self-powered sensors in which the act of sensing and the generation of electrical signals are inherently coupled, enabling simplified architectures and reduced energy overhead [22,23].

Photonic sensing modalities further offer distinct advantages for wearable applications that extend beyond their compatibility with energy harvesting [24]. Optical techniques enable non-contact or minimally invasive interrogation of tissue, allowing access to parameters such as blood oxygenation, glucose levels, and molecular biomarkers with high specificity [25,26]. The spectral tunability of light sources and detectors permits selective targeting of absorption or emission features, while advances in nanophotonic structures and metamaterials have enhanced light–matter interactions at reduced scales [27]. Additionally, optical systems can be engineered to operate across a wide dynamic range of illumination conditions, from low-light indoor environments to direct sunlight, which is particularly advantageous for self-powered operation [28]. These characteristics position photonic sensors as key enablers of multifunctional wearable systems that integrate sensing, communication, and energy harvesting within a unified platform.

To ensure consistent terminology throughout this review, the considered systems are classified according to the source and extent of operational power autonomy. Intrinsically zero-bias photodetectors generate a photocurrent or photovoltage directly under optical excitation without an applied detector bias; however, this condition alone does not imply that signal acquisition, processing, display, or wireless transmission is energy-autonomous. Harvester-assisted systems use an integrated photovoltaic, thermoelectric, triboelectric, or biochemical energy source, with or without storage, to supply the sensing element and associated electronics. Passive optical or colorimetric indicators require no electrical bias and encode the measurand in an optical change, but may require an external camera, reader, or illumination source for quantitative interpretation. Low-bias or near-zero-bias devices are treated separately and are not described as fully self-powered unless their remaining electrical demand is supplied on-device. Here, a photonic sensor is defined as a system in which optical generation, propagation, absorption, emission, or detection is integral to the sensing mechanism; non-optical devices are discussed only when they provide relevant context for energy harvesting, packaging, or wearable system integration [29,30,31,32,33].

To provide a coherent and structured perspective, this review is organized as follows. Section 2 examines the fundamental photophysical mechanisms enabling self-powered operation, including photovoltaic, photothermoelectric, pyroelectric, piezo-phototronic, triboelectric, and photogating effects, with emphasis on their governing physics and inherent trade-offs. Section 3 discusses the material platforms underpinning these mechanisms, covering organic and polymer semiconductors, hybrid halide perovskites, two-dimensional and nanostructured materials, and flexible inorganic systems, with attention to their optoelectronic performance, mechanical compliance, and stability. Section 4 focuses on device architectures, including photovoltaic, photogating-based, waveguide-integrated, fiber/textile-integrated, and ultrathin photonic systems, and analyzes how structural design governs light management and charge transport. Section 5 introduces energy harvesting–sensing co-design strategies, highlighting approaches for integrating energy conversion, storage, and power management within unified platforms. Section 6 addresses the mechanical, environmental, and biophysical constraints that influence device reliability and signal fidelity in wearable operation. Section 7 reviews key sensing modalities and representative applications, particularly in physiological monitoring, environmental sensing, and biochemical detection. Section 8 discusses manufacturing scalability, material sustainability, and packaging strategies required for real-world deployment. Section 9 provides an outlook on emerging directions toward fully autonomous and adaptive wearable photonic systems. Finally, Section 10 concludes the review by summarizing key insights and outlining future perspectives for the development of robust, energy-autonomous wearable photonic technologies.

## 2. Fundamental Photophysical Mechanisms Enabling Self-Powered Operation

The operation of self-powered wearable photonic sensors is fundamentally governed by photophysical mechanisms that directly convert optical, thermal, or mechanically coupled stimuli into electrical signals without external bias [34]. Unlike conventional photodetectors that rely on continuous electrical powering, these systems exploit internal electric fields, polarization effects, or interfacial asymmetries to generate measurable outputs from environmental inputs [35]. In wearable contexts, these mechanisms must function under weak illumination, dynamic deformation, and low-grade thermal or mechanical stimuli, making their efficiency and stability under nonideal conditions critically important [36].

The photovoltaic effect is the most established mechanism enabling self-powered photonic sensing [37]. In this process, incident photons generate electron–hole pairs that are separated by built-in electric fields at p–n junctions, Schottky barriers, or heterointerfaces, producing photocurrent under zero bias [38]. Flexible and low-dimensional materials such as organic semiconductors, halide perovskites, quantum dots, and two-dimensional crystals offer distinct advantages including tunable bandgaps, strong light absorption, and compatibility with bendable substrates [39,40,41,42]. These materials enable thin, lightweight, and conformal devices suitable for on-skin integration. However, their performance is strongly influenced by recombination losses, trap states, and interfacial quality, as well as mechanical durability under repeated strain. In wearable systems, photovoltaic mechanisms often serve a dual function of sensing and energy harvesting, although trade-offs between responsivity and power conversion efficiency must be carefully managed [43].

Photothermal and photothermoelectric effects provide an alternative pathway by converting absorbed light into heat and subsequently into electrical signals [44]. In the photothermoelectric mechanism, spatial temperature gradients induced by nonuniform illumination generate voltage through the Seebeck effect [45]. This approach is particularly effective in materials with high thermoelectric coefficients, including nanostructured conductors, carbon-based films, and hybrid composites. These mechanisms are advantageous for broadband or low-intensity illumination where direct photovoltaic conversion is limited. However, the intermediate thermal process introduces slower response times and lower energy conversion efficiency, with performance strongly dependent on thermal conductivity, heat dissipation, and device geometry. Effective thermal management and localized absorption are therefore essential for optimizing these systems [46].

Pyroelectric and piezo-phototronic effects introduce polarization-mediated coupling between optical, thermal, and mechanical domains [47]. Pyroelectric materials generate transient electrical signals in response to time-varying temperature changes, enabling detection of modulated optical inputs without external bias. This makes them suitable for dynamic sensing but not for steady-state operation [48]. The piezo-phototronic effect, observed in noncentrosymmetric semiconductors such as ZnO and GaN, arises from strain-induced polarization charges that modulate band structures and carrier transport at junctions [49]. In wearable applications, where mechanical deformation is intrinsic, this effect enables simultaneous sensing and performance enhancement by coupling biomechanical stimuli with photocarrier dynamics. However, the magnitude and reproducibility of this effect depend on crystal quality, strain distribution, and interface engineering [50].

Triboelectric and opto-triboelectric mechanisms rely on contact electrification and electrostatic induction to generate electrical output from periodic mechanical motion. When combined with photonic materials, illumination can modulate charge transfer processes, enabling simultaneous optical sensing and energy harvesting [51,52]. These systems are particularly attractive for motion-intensive wearable applications due to their high output voltage and simple structure. Nevertheless, their signals are often nonlinear and sensitive to environmental factors such as humidity and surface condition, which complicates quantitative sensing and long-term stability.

A comparison of these mechanisms reveals inherent trade-offs between energy conversion efficiency, response time, spectral sensitivity, and mechanical adaptability. Photovoltaic systems offer fast response and relatively high efficiency under sufficient illumination, while photothermoelectric systems provide broader spectral response at the cost of slower dynamics [53]. Pyroelectric effects are highly sensitive to temporal changes but are inherently transient, whereas piezo-phototronic and triboelectric mechanisms enable multifunctional coupling with mechanical stimuli but introduce complexity and variability [54]. For wearable self-powered photonic sensors, optimal performance is often achieved through hybrid architectures that combine multiple mechanisms to balance these competing factors under realistic operating conditions [34].

Although the major self-powered photonic sensing mechanisms can be described individually in terms of their underlying physics, direct comparison across platforms remains difficult because their outputs arise from different transduction pathways, operate over different temporal regimes, and are often reported under nonuniform measurement conditions. To provide a more systematic basis for evaluation, Table 1 summarizes the principal self-powered mechanisms relevant to wearable photonic sensors in terms of operating principle, bias requirement, spectral response, representative performance metrics, temporal characteristics, and key limitations. This comparison highlights that self-powered operation is not a single device class but a broader design space spanning junction-driven photovoltaic conversion, thermal and polarization-mediated effects, and mechanically coupled charge-generation pathways, each with distinct implications for sensitivity, speed, stability, and wearable integration.

The numerical ranges in Table 1 are representative values collected from the cited literature and should not be compared as directly equivalent benchmarks because the wavelength, illumination intensity, device area, bias condition, and measurement protocol differ among studies. Responsivity indicates photocurrent or photovoltage generated per optical input, detectivity reflects responsivity together with noise, response time describes temporal behavior, whereas power conversion efficiency and harvested power describe energy-generation performance. These metrics therefore represent different aspects of device operation.

## 3. Material Platforms for Self-Powered Wearable Photonics

The choice of material platform is central to the performance, durability, and scalability of self-powered wearable photonic sensors, as it governs light absorption, charge generation, transport dynamics, and mechanical compatibility. Unlike conventional rigid optoelectronic systems, wearable photonic devices must operate under continuous deformation, exposure to biological fluids, and variable environmental conditions. This imposes stringent requirements on materials, including flexibility, stretchability, environmental stability, and biocompatibility, in addition to strong optoelectronic performance. Recent advances across organic, hybrid, low-dimensional, and inorganic systems have significantly expanded the design space, enabling materials that simultaneously support efficient photophysical processes and mechanical compliance.

### 3.1. Organic and Polymer-Based Semiconductors

Organic and polymer-based semiconductors are among the most promising materials for wearable photonics due to their intrinsic mechanical flexibility, low density, and compatibility with solution processing [58,59]. Their photophysical behavior is dominated by excitonic effects, where tightly bound electron–hole pairs must be dissociated at donor–acceptor interfaces to generate free carriers. Efficient charge separation therefore depends on nanoscale morphology, energy-level alignment, and interfacial engineering. These materials can be engineered into bulk heterojunctions or layered structures that optimize exciton diffusion and carrier extraction. Mechanically, polymers offer excellent compliance and stretchability, enabling conformal integration with skin and textiles [60]. However, their relatively low carrier mobility and susceptibility to environmental degradation remain key challenges. Exposure to oxygen, moisture, ultraviolet radiation, and sweat can induce chemical and structural changes that degrade performance over time. Encapsulation strategies, crosslinking, and the development of intrinsically stable polymer systems are therefore critical for improving long-term reliability [61].

Recent experimental work illustrates how electrode engineering can improve organic wearable photodetectors [62]. A flexible top-illuminated organic photodetector incorporating a germanium-modified silver electrode achieved improved photon transmission, external quantum efficiency, and a photo-to-dark current ratio of approximately 10^4^. The germanium interlayer promoted formation of a continuous ultrathin conductive film with high transmittance and low sheet resistance, while top illumination reduced optical losses associated with the electrode and substrate configuration [62].

**Figure 1:**
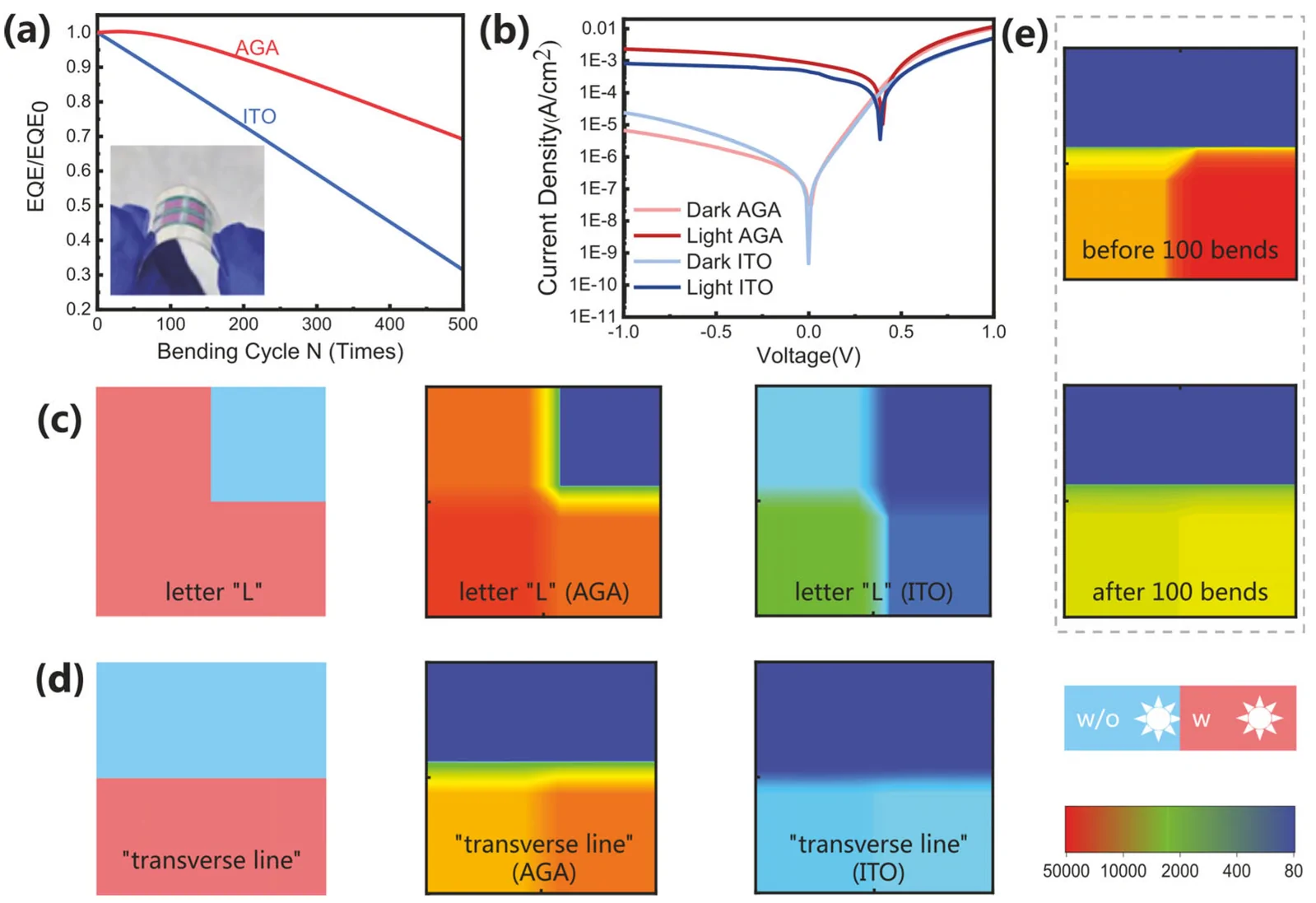
(**a**) External quantum efficiency variation with bending cycles for AGA-based and ITO-based flexible OPDs; insets show device structures, (**b**) current density–voltage characteristics under illumination and dark conditions, (**c**,**d**) imaging of a letter L and a transverse line using 2 × 2 arrays of AGA and ITO devices, with schematics on the left and experimental results on the right, (**e**) imaging of a transverse line using AGA devices before and after 100 bending cycles [62].

The device also retained its performance more effectively during repeated bending than an indium tin oxide-based reference, demonstrating the importance of mechanically compliant transparent electrodes for wearable photonics. However, the gradual degradation of ultrathin metallic electrodes under ambient exposure shows that optical and mechanical optimization must be accompanied by effective encapsulation and material-stability strategies. The corresponding electrical, bending, and imaging results are summarized in Figure 1a–e [62].

### 3.2. Perovskite and Hybrid Halide Materials

Hybrid halide perovskites have attracted significant attention due to their exceptional optoelectronic properties, including high absorption coefficients, long carrier diffusion lengths, and remarkable defect tolerance [63,64]. These characteristics enable efficient light harvesting and charge transport even in thin films, making them highly suitable for self-powered photonic applications. In wearable formats, perovskites offer the potential for high sensitivity and low-light operation, but their practical implementation is hindered by stability and safety concerns. Ion migration under electric field, illumination, or thermal stress can lead to performance drift and hysteresis, while exposure to moisture and oxygen can cause rapid degradation. Encapsulation strategies, compositional engineering, and the development of low-dimensional or all inorganic variants are being explored to address these issues [65]. Additionally, the presence of lead in many high-performance perovskite formulations raises concerns regarding biocompatibility and environmental impact, necessitating the investigation of lead-free alternatives for wearable use [66,67].

Recent advances demonstrate that these materials can be effectively integrated into wearable photonic sensing platforms, particularly through perovskite-based photodetectors. For instance, a printed MAPbI_3_-based photodetector incorporating molecular interface engineering has been reported for real-time and quantitative monitoring of human motion [63]. In this work, 4-acetamidothiophenol (AMTP) was introduced as a passivating agent to regulate perovskite crystallization, resulting in enlarged grain size, reduced defect density, and enhanced carrier lifetime [63]. These improvements directly translated into superior optoelectronic performance, including a high light-to-dark current ratio, fast response time (~12 ms rise and ~18 ms decay), and stable operation under both indoor (~500 lux) and outdoor (~4000 lux) illumination conditions. Quantitative bending tests showed that the AMTP-treated MAPbI_3_ photodetector retained more than 91% of its initial current response after 500 cycles at a bending radius of 7 mm, corresponding to less than 9% degradation, whereas the untreated device exhibited almost complete loss of response [63]. Although this result demonstrates improved mechanical endurance, it does not establish long-term performance under combined wearable stresses. Further assessment should quantify response retention, dark current drift, and changes in response time during simultaneous cyclic deformation and exposure to artificial sweat, controlled humidity, and skin-relevant temperatures, with exposure duration and encapsulation conditions explicitly specified.

**Figure 2:**
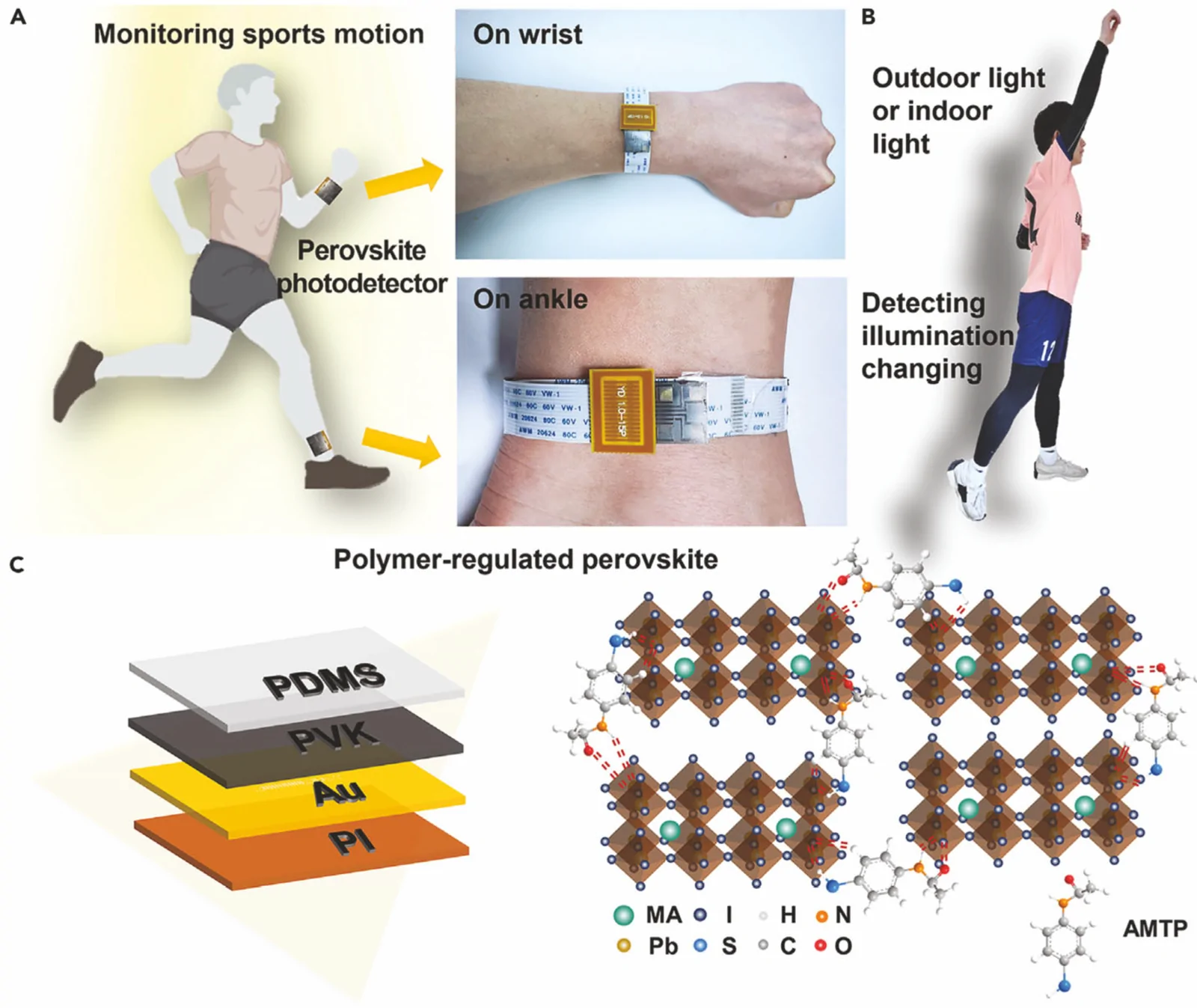
Schematic illustration of a wearable perovskite-based photodetector for real-time and quantitative motion monitoring: (**A**) conceptual representation of the device integrated onto the human body (wrist and ankle) for motion sensing applications, (**B**) operating principle based on the detection of illumination variations induced by body movements under both indoor and outdoor lighting conditions, (**C**) device architecture of the polymer-regulated perovskite photodetector, showing the layered structure and defect-passivation strategy [63].

In this system, limb motion modulates the incident illumination and is converted into an electrical signal by the self-powered perovskite photodetector, enabling non-contact motion monitoring under indoor and outdoor conditions. The AMTP-assisted device design and its wearable operating principle are summarized in Figure 2A–C [63]. However, long-term environmental stability, ion migration, and lead containment remain key barriers to practical wearable deployment.

### 3.3. Two-Dimensional and Nanostructured Materials

Two-dimensional materials and nanostructured systems offer significant potential for self-powered photonic sensing due to their reduced dimensionality and tunable electronic properties [68]. Materials such as transition metal dichalcogenides (e.g., MoS_2_ and WS_2_), graphene, and black phosphorus exhibit strong light–matter interaction, high surface-to-volume ratio, and thickness-dependent band structures [69]. These properties enable precise control over spectral response and carrier dynamics, which are essential for high-performance photodetection. In addition, van der Waals heterostructures formed by stacking different two-dimensional layers provide atomically sharp interfaces with engineered band alignment, facilitating efficient charge separation and transport [70]. The intrinsically thin and flexible nature of these materials further supports their integration into wearable and flexible optoelectronic platforms. Despite these advantages, challenges remain in large-scale synthesis, material uniformity, and environmental stability, particularly for air-sensitive materials such as black phosphorus. Therefore, strategies such as surface passivation, encapsulation, and scalable fabrication techniques are critical for translating these materials into practical devices [71].

An important example highlighting the optoelectronic capabilities of two-dimensional materials is the work by Huo et al., which reports an ultrasensitive all-two-dimensional MoS_2_ phototransistor based on an out-of-plane p–n homojunction [68]. In this device, controlled surface chemical doping forms a vertical p–n junction within few-layer MoS_2_, producing a built-in field that assists spatial separation of photogenerated electron–hole pairs. The device nevertheless operates through photogating, in which trapped photogenerated holes modulate electron transport in the channel and produce high photoconductive gain. Its reported peak responsivity of approximately 7 × 10^4^ A W^−1^ and detectivity of 3.5 × 10^14^ Jones were obtained under finite drain and gate biases, including V_DS_ = 10 V for the peak-performance condition. These results therefore demonstrate the potential of two-dimensional materials for bias-assisted, ultrasensitive photodetection, rather than intrinsically self-powered operation [68].

**Figure 3:**
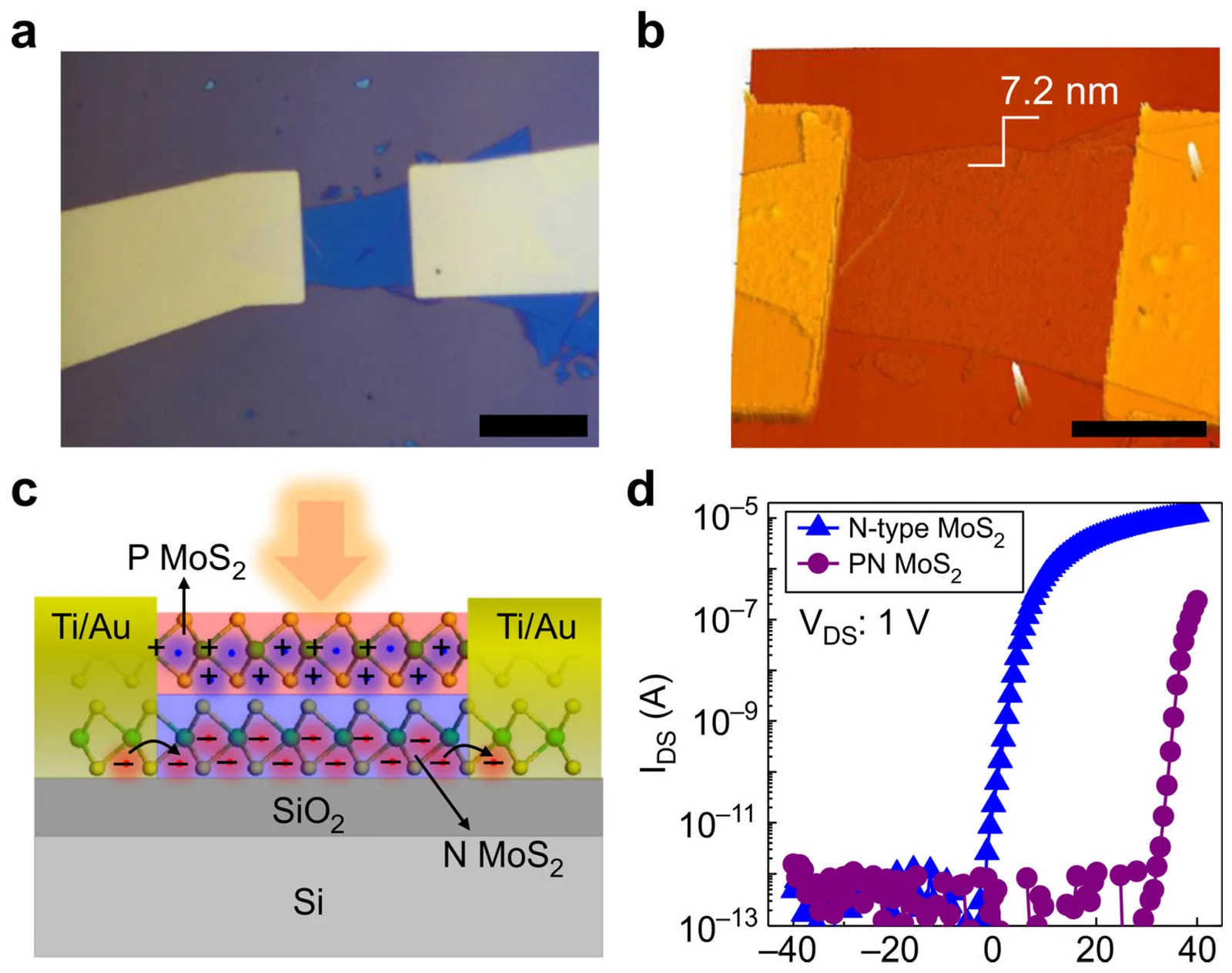
MoS_2_-based phototransistor incorporating an out-of-plane p–n homojunction. (**a**) Optical microscopy image of the few-layer MoS_2_ device with Ti/Au source and drain electrodes (scale bar: 20 µm). (**b**) Atomic force microscopy image indicating a channel thickness of approximately 7.2 nm (scale bar: 10 µm). (**c**) Schematic illustration of the device after surface doping under illumination, showing the formation of a vertical p–n homojunction with p-type MoS_2_ at the top and n-type MoS_2_ beneath; photogenerated holes are confined in the p-type region, while electrons are transported through the n-type channel. (**d**) Transfer characteristics comparing the pristine n-type MoS_2_ device and the doped device, demonstrating a shift in threshold voltage toward positive gate bias, confirming the formation of the p–n junction [68].

The formation of the vertically stacked p–n homojunction, its few-layer MoS_2_ geometry, and the resulting photogating-assisted carrier separation are shown in Figure 3a–d [68]. This example demonstrates the high gain available from two-dimensional photogating devices, but also illustrates their reliance on a biased transport channel and trapped carriers, which can compromise response speed, hysteresis, and long-term stability [68].

Other two-dimensional materials provide complementary capabilities beyond the MoS_2_ photogating example. Black phosphorus offers a narrow but finite bandgap suitable for near-infrared photodetection. Waveguide-integrated BP devices have demonstrated intrinsic responsivities up to 657 mA W^−1^ and response bandwidths exceeding 3 GHz under electrical bias, illustrating their potential for fast optical detection rather than establishing self-powered operation [70]. InSe offers a junction-based alternative: an InSe/InSe_0·82_Te_0·18_ p–n heterojunction exhibited detection over 400–1000 nm and a responsivity of 14.1 mA W^−1^ at 900 nm under zero bias. This result demonstrates how composition-controlled carrier polarity can enable photovoltaic detection without externally biased photoconductive gain [72].

Tellurene extends this materials landscape through anisotropic optical properties that support polarization-sensitive infrared detection. A tellurene-based in-line optical device demonstrated self-driven photodetection at 1550 nm with a responsivity of approximately 0.2 mA W^−1^ [73]. MXenes provide a distinct route through photothermal conversion and conductive interfaces. For example, an asymmetric Ti_3_C_2_T_x_ MXene/ZnO detector achieved zero-bias photothermoelectric detection over 365–808 nm by combining spatially nonuniform heating with differences in Seebeck coefficients [74]. These examples broaden the available sensing mechanisms and spectral ranges, although their reported performance should be compared under matched illumination, bias, and bandwidth conditions. Zero-bias detector operation must also be distinguished from energy autonomy of the complete wearable readout system.

### 3.4. Inorganic Flexible Semiconductors

Inorganic semiconductors such as ZnO, GaN, and related oxide nanostructures offer robust alternatives with high chemical stability, wide bandgaps, and strong resistance to environmental degradation [75]. These materials are particularly well suited for ultraviolet photonic sensing and high-temperature operation, where organic and hybrid systems may fail. Nanowire and thin-film forms enable a degree of mechanical flexibility, especially when integrated onto compliant substrates or embedded within polymer matrices. In addition, many of these materials exhibit piezoelectric properties, enabling coupling between mechanical deformation and photonic response [76,77]. Their advantages include long-term stability, high carrier mobility, and well-established processing methods. However, achieving large-area flexibility without compromising performance remains challenging, and the integration of rigid inorganic materials into soft wearable systems requires careful structural design to manage mechanical stress.

Recent advances have demonstrated that these limitations can be addressed through nanoscale structural engineering and heterostructure design. For example, a self-powered ultraviolet photodetector based on a ZnO-amorphous Ga_2_O_3_ core–shell heterojunction microwire fabricated on a flexible polyethylene terephthalate substrate illustrates how wide-bandgap inorganic semiconductors can be effectively adapted for flexible optoelectronic applications [78]. In this system, ZnO provides a direct wide bandgap and high carrier mobility, while the amorphous Ga_2_O_3_ shell, deposited at low temperature, introduces a larger bandgap, and forms a sharp heterointerface that enhances carrier separation and ultraviolet selectivity. The resulting device operates without external bias due to the built-in electric field of the heterojunction and exhibits high responsivity in the deep-ultraviolet region together with fast and stable photoresponse characteristics. The one-dimensional microwire geometry further contributes to improved sensitivity and mechanical compliance by combining high crystallinity with a large surface-to-volume ratio [78].

**Figure 4:**
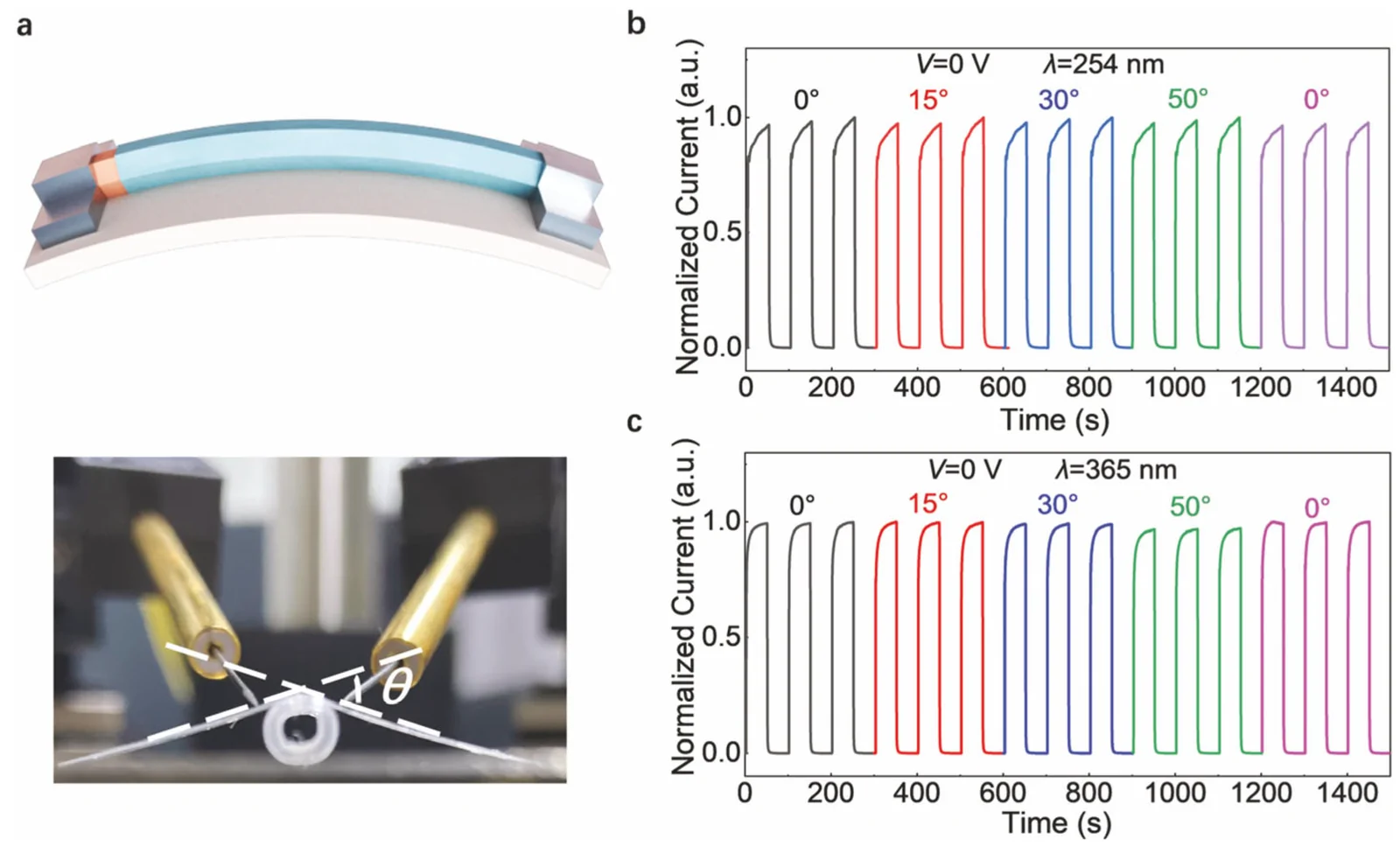
(**a**) Schematic illustration and optical image of the bending test configuration for the flexible ZnO–a-Ga_2_O_3_ core–shell microwire photodetector. Normalized time-dependent photocurrent (I–t) responses measured at 0 V under ultraviolet illumination at (**b**) 254 nm and (**c**) 365 nm for varying bending angles [78].

The mechanical robustness of such inorganic nanostructures is highlighted by the bending experiments shown in Figure 4a–c [78]. In Figure 4a, the schematic illustration and corresponding optical image present the flexible device configuration and the method used to apply mechanical deformation, where the bending angle is controlled by adjusting the relative position of the electrodes, introducing strain along the microwire. Figure 4b shows the normalized time-dependent photocurrent response under 254 nm ultraviolet illumination at different bending angles ranging from the flat state to approximately 50 degrees and back. The nearly identical current switching profiles across all bending conditions indicate that the device maintains stable photoresponse without noticeable degradation. Similarly, Figure 4c presents the photocurrent behavior under 365 nm illumination, again demonstrating consistent response amplitude and switching stability regardless of mechanical deformation. The preservation of electrical and photonic performance across varying bending states confirms that the heterojunction interface and carrier transport pathways remain intact under strain. This behavior highlights the effectiveness of combining one-dimensional nanostructures with amorphous oxide shells to accommodate mechanical stress, providing a viable pathway for integrating rigid inorganic semiconductors into flexible and wearable optoelectronic systems [78].

Beyond the active semiconductor, substrate selection determines the accessible illumination geometry and fabrication temperature. Polyethylene terephthalate (PET) provides an inexpensive, lightweight, bendable support compatible with low-temperature coating and printing, although thermal shrinkage constrains subsequent annealing [79]. Its visible transparency should not be interpreted as deep-ultraviolet transparency; when the substrate absorbs the target wavelength, illumination should reach the active layer from the device side. Poly(vinylidene fluoride) (PVDF) offers a flexible alternative with good chemical resistance, and appropriately prepared PVDF films have demonstrated sufficient UVC transmission for wearable, self-powered perovskite photodetectors [80]. However, transmission depends on film thickness and morphology, while solvent exposure and heating must be controlled to preserve substrate integrity.

Commercial ultrathin flexible glass, such as Corning Willow Glass, combines bendability with greater thermal and dimensional stability than these polymer supports, enabling a wider range of deposition and annealing processes. A reported 100 µm thick Willow Glass substrate transmitted approximately 60% of incident light at 254 nm, demonstrating its potential for illumination through the substrate in UVC photodetectors [81]. Corning specifies processing temperatures up to 500 °C for the glass itself, although the complete device stack may impose a lower limit. Its brittle nature and limited tensile strain nevertheless require controlled bending and protected edges. Consequently, polymer films are attractive for lightweight wearable patches, whereas ultrathin glass is better suited to gently curved modules requiring greater processing stability. For all three substrates, bendability alone does not establish stretchability or reliable conformity to moving skin.

## 4. Device Architectures for Self-Powered Photonic Sensing

The performance and practicality of self-powered wearable photonic sensors are strongly determined by device architecture, where material properties, photophysical mechanisms, and system-level considerations converge. Beyond the choice of active materials, architectural design governs how light is captured, how carriers are generated and transported, and how electrical signals are extracted under zero-bias conditions. In wearable systems, these architectures must additionally accommodate mechanical flexibility, conformal integration with skin or textiles, and compatibility with scalable fabrication [82]. As a result, device design is not merely a structural consideration but a critical factor that defines sensitivity, energy efficiency, and robustness under real-world conditions.

### 4.1. Photovoltaic Photodetectors

Photovoltaic photodetectors represent the most direct architectural implementation of self-powered photonic sensing, relying on built-in electric fields to separate photogenerated carriers without external bias [83]. These devices are typically based on p–n junctions, Schottky contacts, or heterostructures that create internal potential gradients for charge separation. This operating principle enables low-noise detection and inherently energy-efficient device functionality, making photovoltaic photodetectors particularly attractive for autonomous and wearable sensing platforms. In wearable formats, thin-film and flexible photovoltaic architectures are widely used due to their low-power consumption, fast response, and compatibility with lightweight substrates [84]. In recent years, significant advances have been made in the development of such systems, incorporating materials including organic semiconductors, perovskites, and two-dimensional materials deposited on mechanically compliant substrates. These material platforms support large-area fabrication while maintaining mechanical robustness under bending and deformation [85]. The design of these devices must balance absorption efficiency with carrier extraction, often requiring optimization of layer thickness, interface quality, and electrode transparency. While increasing the thickness of the active layer can enhance optical absorption, it may also lead to increased recombination losses and reduced carrier transport efficiency. Consequently, interface engineering, such as defect passivation and energy-level alignment, plays a crucial role in improving charge separation and transport across device interfaces.

Advanced architectures incorporating tandem structures or graded band alignment can further enhance performance by improving spectral utilization and reducing recombination losses [86]. Tandem configurations enable the stacking of materials with complementary bandgaps to extend spectral sensitivity, whereas graded band structures facilitate smoother carrier transport and suppress interfacial recombination. These strategies collectively contribute to enhanced responsivity, faster response times, and improved operational stability [85].

Flexible photovoltaic architectures extend beyond planar substrates to fiber-shaped devices and photovoltaic textiles. In coaxial designs, photoactive and charge-transport layers surround a conductive fiber core, while a light-transmitting outer electrode completes the device, enabling integration into woven structures. Qiu et al. demonstrated a coaxial perovskite solar fiber with a wrapped carbon nanotube electrode, achieving a power conversion efficiency of 3.3% and retaining approximately 95% of its initial efficiency after 50 bending cycles [87]. An alternative approach fabricates photovoltaic devices directly on suitably prepared textile substrates. Jeong et al. developed textile-based polymer solar cells protected by an encapsulation barrier incorporating a SiO_2_–polymer composite capping layer. Integrated photovoltaic and light-emitting modules exhibited little performance change after 20 washing cycles of 10 min each under the reported test conditions [88]. Adapting these architectures for photonic sensing requires uniform coatings and reliable electrical contacts under deformation. Although these demonstrations establish photovoltaic energy harvesting in wearable formats, their suitability for direct zero-bias photodetection additionally requires evaluation of responsivity, noise, response time, and signal stability under the intended operating conditions.

### 4.2. Photonic Sensors Based on Photogating Effects

Photogating-based devices offer an alternative architecture that leverages charge trapping and internal gain mechanisms to enhance sensitivity under low-light conditions [89]. In these systems, photogenerated carriers become trapped in localized states, modulating the conductivity of a channel material and effectively amplifying the photoresponse. This approach is particularly advantageous for detecting weak optical signals, as it can produce high responsivity even in self-powered or near-zero-bias operation [90]. However, the presence of trap states introduces trade-offs, including slower response times and potential signal hysteresis. In wearable applications, careful control of trap density and distribution is required to achieve stable and repeatable performance, especially under varying environmental and mechanical conditions [91]. These characteristics are exemplified by graphene–quantum dot hybrid photodetectors, where the combination of a high-mobility transport channel and a strongly absorbing sensitizer enables efficient photogating with large gain [61].

In such devices, incident light is absorbed in the quantum dot layer, generating electron–hole pairs, after which one carrier type is transferred to the graphene channel while the opposite carriers remain trapped within the quantum dots [61]. The trapped charges act as a local electrostatic gate, modulating the conductivity of graphene and producing significant photoconductive gain. As a result, these devices exhibit exceptionally high responsivity, exceeding 10^5^ A W^−1^ under low illumination, along with broadband spectral sensitivity extending from the visible to the near-infrared region. At the same time, the high carrier mobility of graphene ensures efficient charge transport and relatively low noise, enabling reliable signal extraction even under weak-light conditions. The architecture is also inherently compatible with flexible substrates, allowing integration into wearable systems that maintain stable performance under mechanical deformation.

**Figure 5:**
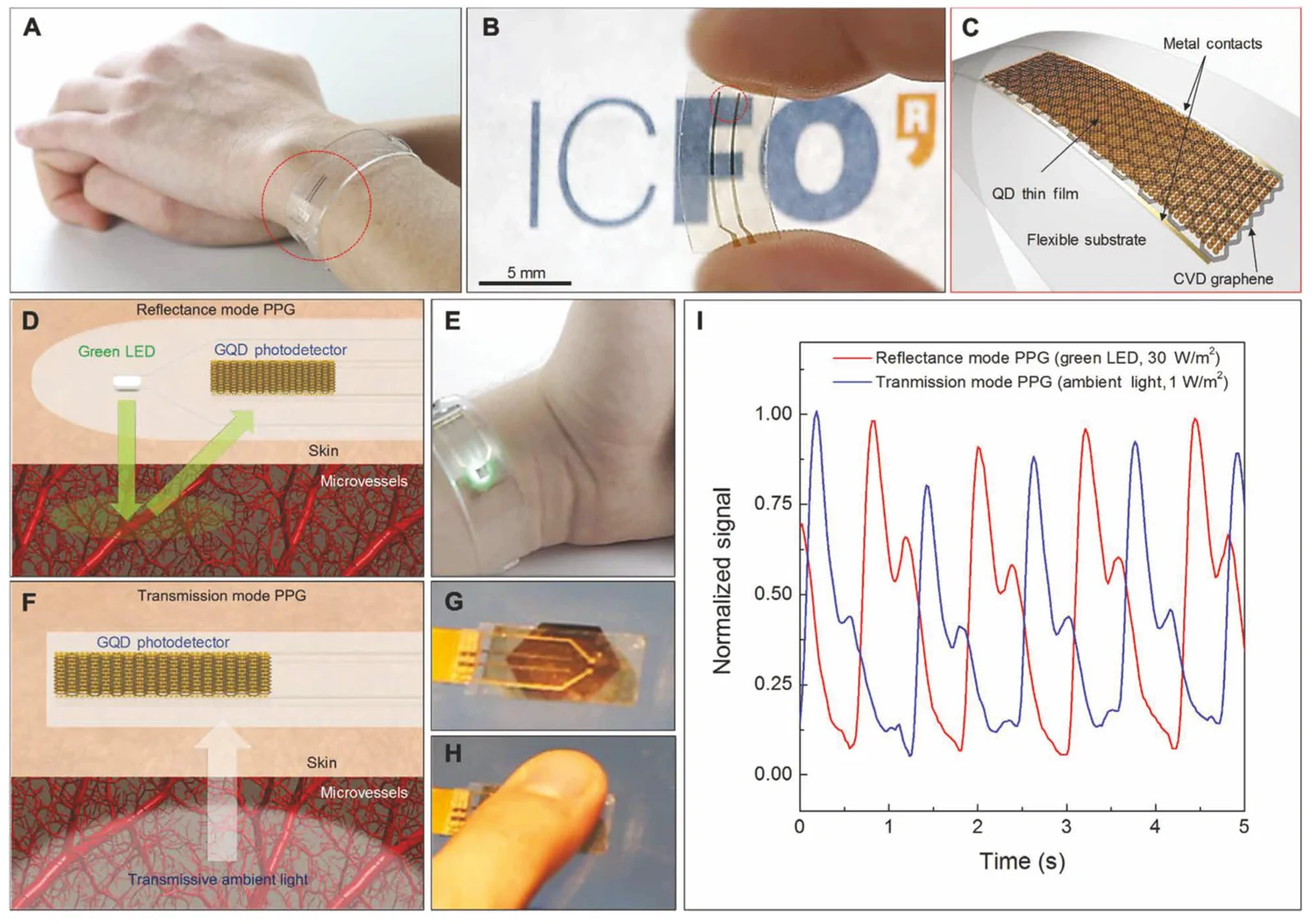
Graphene–quantum dot photodetector for wearable health monitoring: (**A**) flexible and transparent device integrated into a wrist-mounted heart rate monitoring system; (**B**) magnified view of the photodetector showing a graphene channel on a polymer substrate coated with a thin PbS quantum dot layer; (**C**) schematic illustration of the device structure combining graphene and quantum dots; (**D**,**E**) reflectance-mode photoplethysmography where backscattered light modulated by blood volume changes is detected at the wrist; (**F**) schematic of transmission-mode operation; (**G**,**H**) wearable configuration for transmission-mode sensing; (**I**) normalized photoplethysmography readings for transmission and reflectance modes of operation [61].

The device structure and wearable implementation are illustrated in Figure 5A–G [61]. Figure 5A shows a flexible and transparent photodetector integrated into a wrist-mounted platform for heart rate monitoring, demonstrating conformal contact with the skin. Figure 5B provides a magnified view of the active device, where a graphene channel on a polymer substrate is fully covered by a thin quantum dot layer, highlighting both mechanical flexibility and optical transparency [61]. Figure 5C presents the device architecture, illustrating the integration of the graphene transport layer with the quantum dot absorber, which enables photogating through trap-assisted charge transfer. Figure 5D illustrates the principle of reflectance-mode photoplethysmography, where variations in blood volume modulate the intensity of backscattered light detected at the skin surface [61]. Figure 5E shows the corresponding wearable bracelet configuration used for wrist-based physiological monitoring. Figure 5F presents the transmission-mode sensing principle, in which light passing through tissue is modulated by the cardiac cycle before reaching the detector. Figure 5G shows a compact wearable implementation of this mode, demonstrating integration with portable platforms. Together, these results highlight how photogating-based device architectures can be translated into flexible, highly sensitive, and low-power wearable photonic sensors for real-time physiological monitoring [61].

### 4.3. Waveguide-Integrated Wearable Photonic Structures

Waveguide-integrated architectures enable controlled propagation and manipulation of light within the device, enhancing interaction between optical signals and sensing elements [92]. Flexible optical waveguides can be embedded in polymer substrates or elastomeric layers to direct light to specific regions, improve signal collection efficiency, and reduce losses due to scattering or misalignment [8,93,94]. These structures are particularly useful for multiplexed sensing, where multiple sensing sites are addressed along a single optical path. In wearable systems, waveguide integration allows decoupling of the sensing region from the detection or energy harvesting unit, enabling more sophisticated designs while maintaining compact form factors [95]. Challenges include maintaining optical confinement under deformation and minimizing losses at interfaces and bends [96].

The study reported in Leal-Junior et al. demonstrates a bioinspired multifunctional flexible optical waveguide sensor in which optical signal transmission and mechanical functionality are co-integrated within a single polymeric structure [93]. A core–cladding waveguide configuration is realized using a photocurable resin as the core and polydimethylsiloxane as the cladding, enabling efficient light guiding through total internal reflection while maintaining high flexibility. The refractive index contrast between these materials supports stable optical confinement, whereas their complementary mechanical properties allow the structure to sustain significant deformation without compromising optical performance. The photocurable resin provides structural rigidity, while the elastomeric PDMS contributes to large strain tolerance, which is essential for wearable applications. The device exhibits low optical loss and stable operation over a frequency range relevant to human motion, confirming the feasibility of integrating optical waveguiding with mechanically compliant substrates [93].

A significant finding of this work is the realization of distributed and multiplexed sensing through a network of interconnected waveguide branches. Mechanical perturbations applied to different regions of the structure induce localized strain variations that modulate the transmitted optical intensity, enabling simultaneous detection of force magnitude and spatial location. The sensor achieves high sensitivity with force detection in the micro-Newton range and spatial resolution at the sub-millimeter scale, demonstrating effective optomechanical coupling within flexible waveguides. In addition, the integration of the light source within the structure enables separation of the sensing regions from the detection units while preserving a compact and lightweight form factor. The system is validated through wearable applications including hand motion tracking, trunk orientation monitoring, and respiration rate measurement, highlighting its capability for real-time physiological sensing in soft and conformable platforms [93].

**Figure 6:**
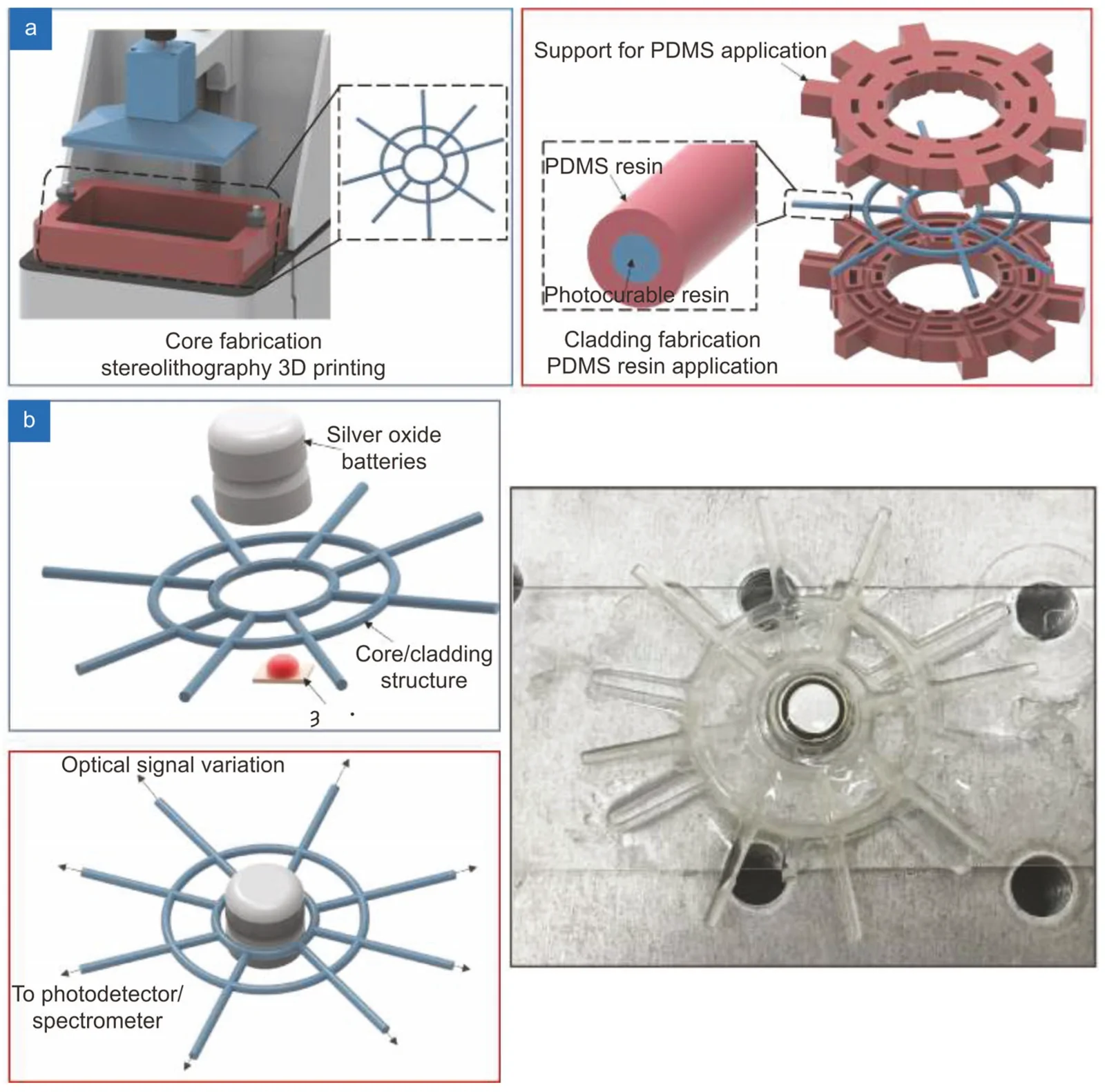
(**a**) Schematic illustration of the fabrication process of the core–cladding waveguide structure; (**b**) integration of the battery and micro-light-emitting diode within the device, serving as the internal light source and central proof mass, along with a photograph of the fabricated BioMFOS [93].

The fabrication and integration strategy of the waveguide system is illustrated in Figure 6. In Figure 6a, the formation of the core–cladding waveguide structure is achieved through stereolithography-based three-dimensional printing of the photocurable resin, where ultraviolet exposure defines the geometry of the waveguide network with precise control. The printed core is subsequently embedded within a PDMS layer that serves as the cladding, providing both optical confinement and mechanical compliance. This approach enables the realization of a continuous and flexible waveguide network capable of guiding light across multiple sensing branches, which is particularly advantageous for distributed sensing in wearable systems. Figure 6b depicts the integration of optical and electronic components within the structure. A micro-light-emitting diode is positioned at the center of the device and acts as the internal light source, while compact batteries are incorporated to supply power. The emitted light is coupled into the surrounding waveguide network and propagates along the radial elements, where mechanical deformation alters the optical path and modulates the transmitted signal. The assembled device demonstrates a compact and lightweight configuration suitable for wearable deployment. However, because the micro-light-emitting diode and associated electronics are supplied by compact batteries, this platform is not an energy-autonomous or fully self-powered photonic sensor. It is included here as a transferable example of waveguide integration, optical routing, and mechanically compliant wearable system design [93].

### 4.4. Fiber-Based and Textile-Integrated Photonic Sensors

Optical fibers and photonic structures integrated into textiles provide a natural platform for wearable sensing due to their inherent flexibility and compatibility with fabrics [97]. Fiber-based sensors can guide light over long distances with low loss, enabling distributed sensing across large areas of the body. Textile integration allows seamless incorporation into clothing, supporting applications such as motion monitoring, physiological sensing, and environmental detection [98]. In self-powered configurations, fibers can be combined with photovoltaic or triboelectric elements to enable energy harvesting alongside sensing. However, maintaining optical performance under bending, stretching, and repeated washing remains a key challenge, requiring robust materials and protective coatings [99].

Recent advances in fiber-integrated smart textiles have demonstrated the feasibility of embedding highly flexible polymer optical fibers within fabric structures to achieve multiparameter sensing. In particular, polymer optical fibers fabricated through light polymerization spinning exhibit significantly reduced Young’s modulus and enhanced stretchability, enabling their integration into textiles without altering the mechanical properties of the fabric or restricting user movement [100]. These systems utilize intensity variation in transmitted light as the sensing mechanism, allowing the detection of temperature, applied force, and mechanical deformation with high linearity and repeatability. Furthermore, the implementation of multiplexing techniques enables quasi-distributed sensing along a single fiber, allowing multiple sensing points to be monitored simultaneously across different regions of the body. Experimental demonstrations have shown the capability of such textile systems to monitor human activities, including walking, sitting, and squatting, through signal processing approaches such as principal component analysis, highlighting their potential for wearable health monitoring and human motion analysis [100].

**Figure 7:**
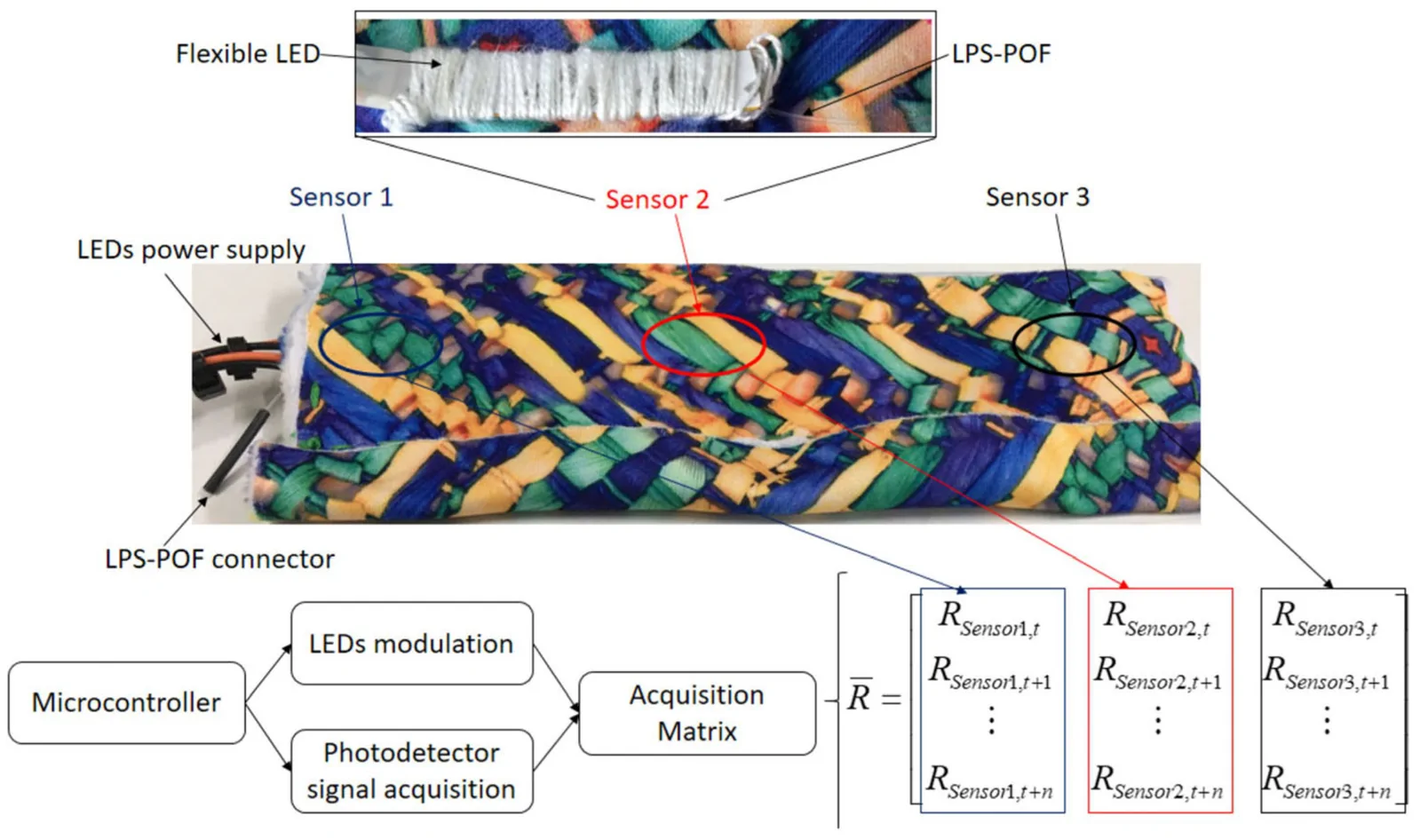
Schematic illustration and photographic image of the proposed multifunctional smart textile, including the data acquisition matrix representing the sensor responses [100].

The structural configuration of these textile-integrated photonic sensors is illustrated by the embedded fiber architecture (Figure 7), where the optical fiber and associated optoelectronic components are integrated within layers of fabric to maintain both functionality and wearability [100]. In this configuration, a polymer optical fiber is embedded within the textile and coupled with flexible light-emitting diodes that act as distributed light sources, while photodetectors are connected at the fiber ends to capture variations in transmitted optical power. The spatial arrangement of multiple light sources along the fiber enables localized sensing regions, where each segment of the fiber responds to external stimuli such as pressure, temperature, or bending [100]. The modulation of individual light sources in a time-sequenced manner allows the system to distinguish signals from different sensing locations, effectively creating a multiplexed sensing network within a single fiber. This architecture supports real-time data acquisition and enables scalable sensing across the textile without significantly increasing system complexity. The integration of these components within a flexible fabric substrate preserves textile conformability while enabling reliable signal transmission under mechanical deformation. However, because the light-emitting diodes and photodetectors are not supplied by a clearly demonstrated on-device energy source, this system is discussed as a transferable example of textile-integrated photonic sensing rather than a fully self-powered platform [100].

### 4.5. Transparent and Imperceptible Device Designs

For many wearable applications, user comfort and esthetic considerations are as important as technical performance [101,102]. Transparent and imperceptible device architectures aim to minimize visual and physical intrusion by using ultrathin, lightweight, and optically transparent materials [103]. These designs enable unobtrusive integration on skin or within clothing, improving user acceptance and enabling continuous monitoring. Achieving transparency often involves the use of transparent conductive electrodes, thin active layers, and substrate materials with matched refractive indices. At the same time, these devices must maintain sufficient optical absorption and electrical conductivity to function effectively, which introduces design trade-offs. Advances in nanomaterials, ultrathin films, and soft electronics are enabling increasingly invisible photonic systems that retain high performance while enhancing wearability [44,104].

Building on these design principles, recent advances in ultrathin organic optoelectronic systems further demonstrate how imperceptibility can be realized in practical wearable devices without necessarily relying on full optical transparency [105]. In particular, the development of micrometer-scale, skin-conformable photonic systems highlight that reducing device thickness and mechanical mismatch is equally critical for achieving truly unobtrusive integration with the human body. For example, ultraflexible self-powered photonic skins that integrate light emission, sensing, and energy harvesting components within a single platform have been shown to conform intimately to the skin while enabling continuous physiological monitoring. Such systems illustrate that imperceptibility can be achieved through extreme thinness and mechanical compliance, complementing strategies based on transparent materials [105].

**Figure 8:**
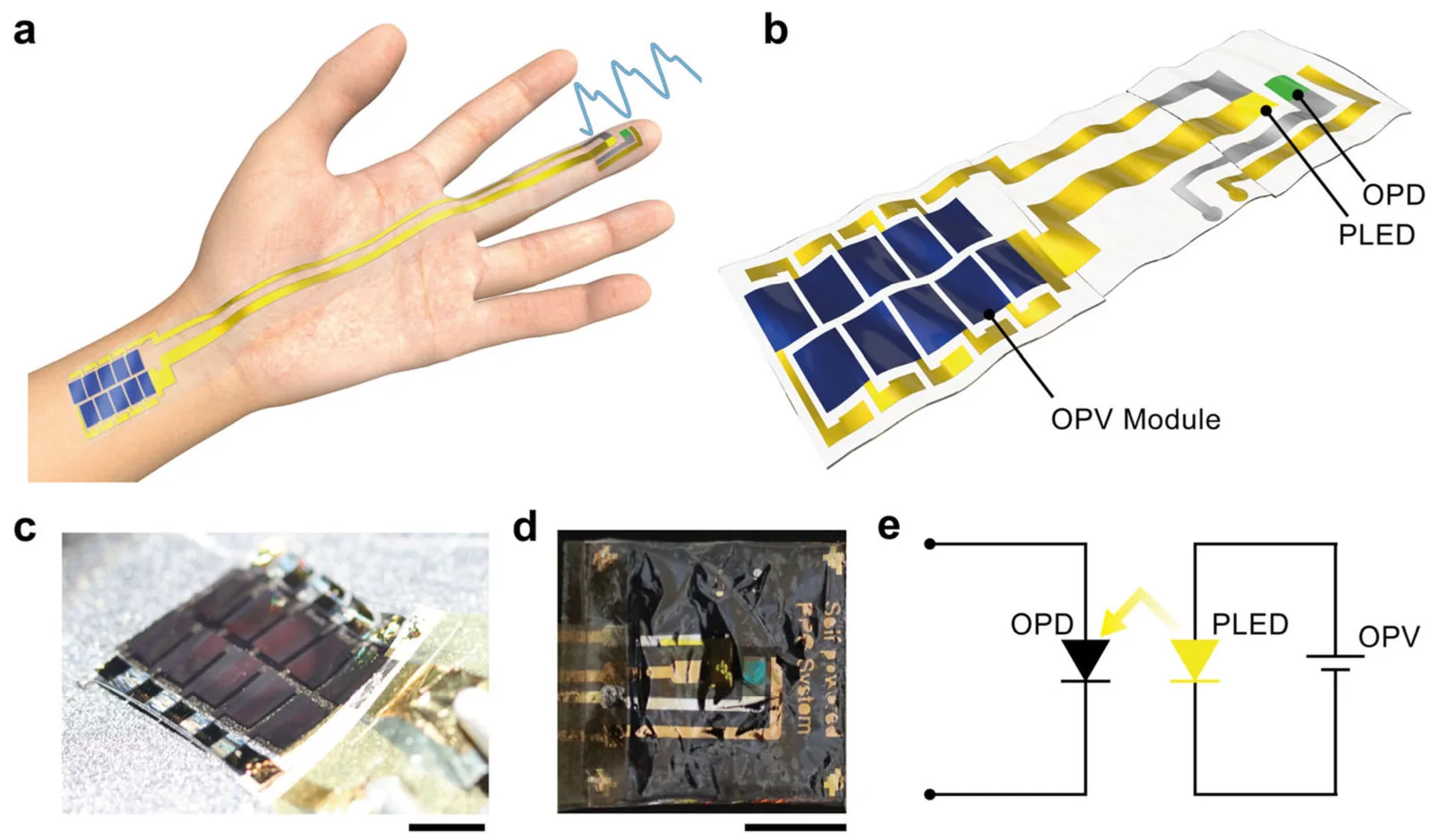
(**a**) Schematic illustration of the ultraflexible, self-powered photoplethysmogram sensor conformally integrated on human skin, where an organic photovoltaic module harvests incident light and supplies electrical energy to drive a polymer light-emitting diode and an organic photodetector; (**b**) structural schematic of the integrated sensing system showing the configuration of the polymer light-emitting diode, organic photodetector, and organic photovoltaic module interconnected within a flexible platform; (**c**) photograph of the ultraflexible organic photovoltaic module under one-sun illumination, demonstrating its power generation capability; (**d**) top view photograph of the fully integrated ultraflexible self-powered photoplethysmogram sensor, highlighting its compact and film-like architecture; (**e**) electrical circuit diagram of the self-powered sensing system illustrating the direct coupling between the photovoltaic module, light-emitting diode, and photodetector [105].

As illustrated in Figure 8, the overall concept and implementation of such a system are presented through multiple complementary views. In Figure 8a, a schematic depiction shows the ultraflexible device laminated directly onto a human hand, where the photovoltaic module harvests ambient light and supplies power to the integrated light-emitting diode and photodetector. This representation emphasizes both the conformal attachment to the skin and the ability to perform continuous, noninvasive monitoring of physiological signals through optical interaction with underlying tissue [105]. Figure 8b provides a structural schematic of the integrated system, illustrating the arrangement and interconnection of the ultraflexible polymer light-emitting diode, organic photodetector, and organic photovoltaic module. These components are linked via thin, flexible conductive pathways, forming a unified platform in which energy harvesting, light emission, and signal detection are seamlessly combined while maintaining mechanical compliance. In Figure 8c, a photograph of the ultraflexible photovoltaic module under illumination demonstrates its practical functionality and confirms its capability to generate electrical power from incident light, while also highlighting its thin and lightweight form factor. Figure 8d presents a top view photograph of the complete photoplethysmogram sensor, showing the compact integration of all functional components on a flexible substrate, with a film-like appearance that supports unobtrusive attachment to the skin. Lastly, Figure 8e illustrates the electrical circuit of the self-powered sensing system, clarifying the operational mechanism in which the photovoltaic module directly powers the light-emitting diode and the photodetector converts reflected optical signals associated with blood flow into electrical outputs. This integrated configuration enables continuous physiological monitoring without the need for external power sources [105].

The architectural design of self-powered wearable photonic sensors defines how photophysical processes are translated into measurable electrical signals under zero- or near-zero-bias conditions, while simultaneously satisfying constraints imposed by mechanical compliance and system integration. Unlike purely mechanism-based classifications, device architectures determine the spatial organization of active materials, optical pathways, and charge-transport interfaces, thereby governing signal extraction efficiency, noise characteristics, and robustness under deformation. In wearable formats, these architectures must also accommodate conformal contact with soft biological surfaces, maintain optical coupling under strain, and enable scalable fabrication on flexible substrates. To provide a systematic framework for comparison, Table 2 summarizes the principal device architectures employed in self-powered wearable photonic sensing, highlighting their representative structures, underlying transduction mechanisms, carrier transport physics, operational regimes, and key performance trade-offs. It is intended to compare architecture classes rather than provide directly benchmarked, study-by-study performance data, because reported demonstrations use non-matched optical, electrical, mechanical, and system-level test conditions.

## 5. Energy Harvesting–Sensing Co-Design

The realization of truly autonomous wearable photonic systems requires a shift from treating sensing and energy harvesting as independent modules toward an integrated co-design paradigm in which both functions are intrinsically coupled [111]. In conventional architectures, energy harvesting units are often appended to sensing components, leading to inefficiencies in power transfer, increased system complexity, and suboptimal utilization of available energy [112,113]. In contrast, energy harvesting–sensing co-design seeks to align photophysical processes, material properties, and device architectures such that the same incident stimulus contributes simultaneously to signal generation and energy conversion [114]. This approach is particularly important in wearable applications, where the available energy from ambient light, body heat, and motion is limited and highly variable. By co-optimizing these pathways, it becomes possible to reduce energy overhead, improve system efficiency, and enable continuous operation under realistic conditions.

### 5.1. Co-Optimization of Sensing and Energy Harvesting

Co-optimization of sensing and energy harvesting requires balancing sensing performance with energy conversion efficiency rather than optimizing them independently [115]. In photonic systems, this is achieved by tuning band structure, absorption, and carrier transport so that photogenerated carriers contribute to both signal generation and power output. Design trade-offs are inherent: increasing absorption thickness improves energy conversion but introduces recombination losses and slower response, while optimizing electrode selectivity affects linearity and noise. Effective device design therefore requires a system-level approach that jointly considers responsivity, power conversion efficiency, response time, and stability. Architectures based on asymmetric junctions, graded band structures, and multifunctional heterointerfaces are particularly promising, as they enable coordinated control over carrier generation, separation, and transport [116].

**Figure 9:**
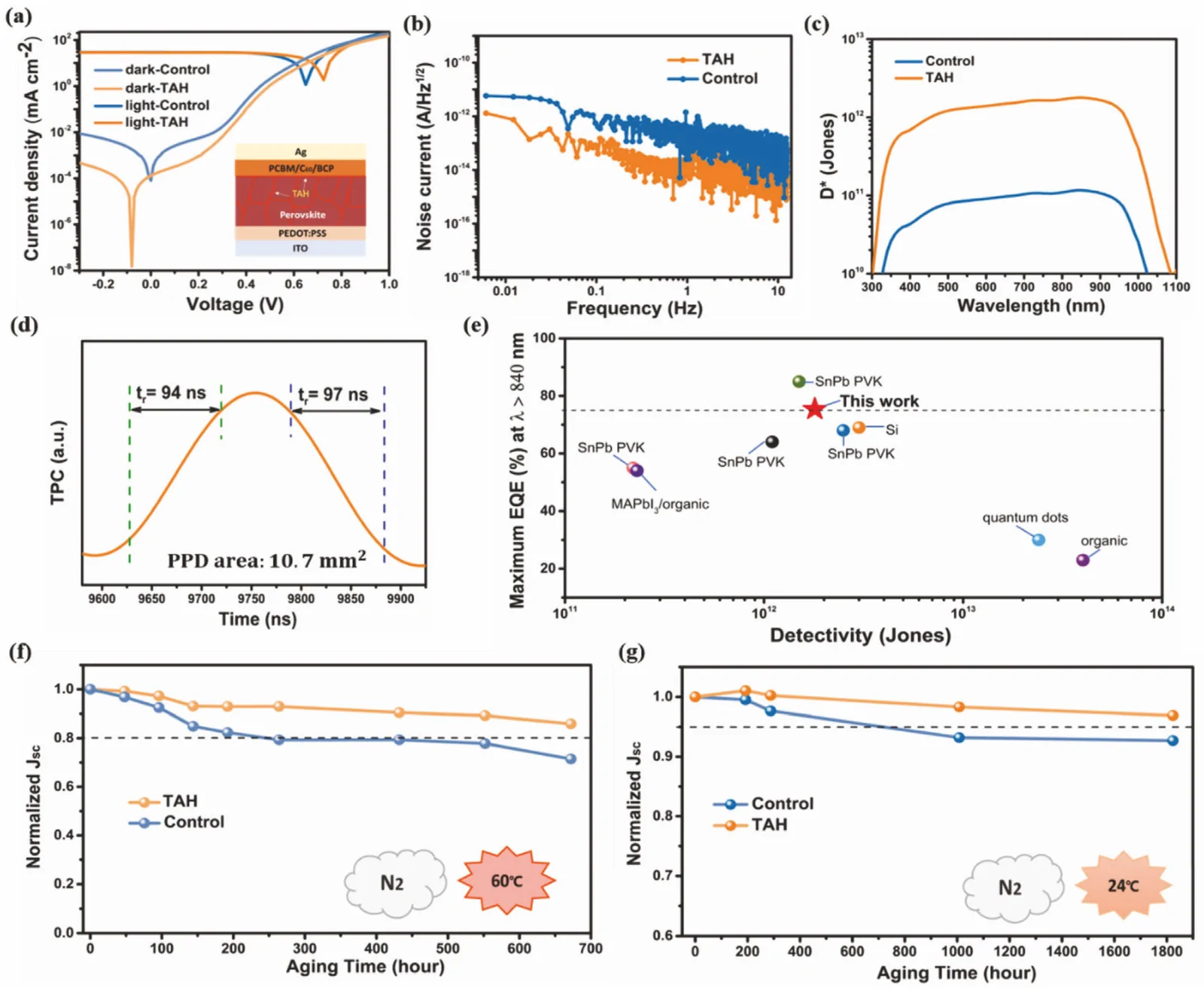
Influence of functional passivating antioxidant (FPA) treatment on the performance and stability of mixed Sn–Pb perovskite photodetectors: (**a**) dark and illuminated current density–voltage (J–V) characteristics of the mixed Sn–Pb (MSP) devices, with the inset showing the inverted device architecture; (**b**) noise current spectra of the MSP photodetectors measured at zero bias; (**c**) spectral detectivity (D*) of the MSP devices under zero-bias operation; (**d**) temporal response characteristics, showing rise and decay times at zero bias; (**e**) correlation between maximum external quantum efficiency (EQE) for wavelengths above 840 nm and detectivity at zero bias; (**f**) thermal stability and (**g**) long-term shelf stability of the mixed Sn–Pb photodetectors [117].

This co-optimization approach is illustrated by self-powered mixed tin–lead perovskite photodetectors in which functional passivation reduces trap-assisted recombination and low-frequency noise, improving carrier extraction, detectivity, and response speed at zero bias [117]. The electrical, noise, spectral-detectivity, temporal response, and stability results are compiled in Figure 9a–g [117]. This example shows that defect control can improve several sensing metrics simultaneously; however, independent evidence of energy harvesting efficiency, long-term mechanical reliability, and operation under realistic wearable illumination remains necessary before broader system-level claims can be made [117].

### 5.2. Spectral Selectivity and Ambient Light Utilization

Efficient use of ambient light is central to self-powered photonic operation, but the spectral composition of available illumination varies widely between indoor and outdoor environments. Co-design strategies must therefore account for spectral selectivity to ensure that devices can both harvest energy and perform sensing under diverse lighting conditions [118]. Materials and structures can be engineered to selectively absorb specific wavelength ranges relevant to the sensing task while simultaneously converting other portions of the spectrum into electrical power [119]. For instance, broadband absorbers may be combined with wavelength-selective filters or layered architectures that separate sensing and harvesting functions spectrally. In wearable applications such as physiological monitoring, where specific wavelengths are required for interaction with biological chromophores, balancing spectral selectivity with overall energy capture is particularly critical [120].

Recent advances in photonic device engineering have demonstrated that spectral selectivity can be intrinsically achieved through material and structural design rather than relying on external optical filters. In particular, self-powered photodetectors based on hybrid and quasi-tandem architectures enable dual-mode operation, exhibiting narrowband wavelength-selective detection alongside broadband absorption for energy harvesting within a single device [121]. Such behavior is enabled by carefully engineered band alignment and carrier transport pathways, allowing selective extraction of photogenerated carriers corresponding to targeted spectral regions while suppressing unwanted background signals. This approach provides a versatile platform for adapting device functionality to varying illumination environments, particularly in scenarios where ambient light intensity and spectral distribution are not constant [121].

Layered and heterostructured device configurations further enhance spectral control by spatially separating optical absorption and charge collection processes. By combining materials with complementary bandgaps, these architectures enable spectral splitting within a single device, where high-energy photons are preferentially utilized for sensing while lower energy photons contribute to power generation [122]. This strategy improves overall energy utilization efficiency while preserving detection specificity. In addition, interfacial engineering and thickness optimization can be used to tune optical interference effects and carrier recombination dynamics, enabling filter-free narrowband response with high signal-to-noise ratio [123]. Such designs are particularly advantageous for compact and wearable systems, where minimizing optical components is essential for mechanical flexibility and integration. Furthermore, integrated photonic systems designed for ambient light utilization demonstrate that spectral response can be tuned to match specific illumination conditions, including low-intensity indoor lighting. Devices engineered with optimized absorption profiles exhibit enhanced performance under artificial light sources such as LEDs and fluorescent lamps, while maintaining functionality under broadband solar illumination [124]. Collectively, these advances illustrate the critical role of co-designed material systems and device architectures in achieving simultaneous spectral selectivity and efficient ambient light utilization, enabling the development of multifunctional, self-powered photonic sensors for next-generation wearable applications.

### 5.3. Hybrid Harvesting (Solar + Thermal + Mechanical)

Relying on a single energy source is often insufficient for maintaining continuous operation in wearable environments, where light, temperature gradients, and mechanical motion vary over time. Hybrid energy harvesting approaches that combine multiple mechanisms, such as photovoltaic, thermoelectric, and triboelectric processes, offer a more robust solution. By integrating these mechanisms within a unified platform, it is possible to harvest energy from complementary sources and maintain functionality under a broader range of conditions. For example, solar energy may dominate under outdoor illumination, while thermal gradients between skin and environment or biomechanical motion can provide supplementary power indoors or during low-light periods. The challenge in hybrid systems lies in efficiently combining outputs with different voltage, current, and temporal characteristics without introducing excessive complexity or losses.

### 5.4. Energy Storage Integration (Microsupercapacitors)

Given the intermittent and variable nature of harvested energy, on-device energy storage is essential for stabilizing power supply and enabling continuous sensing and data processing. Microsupercapacitors have emerged as attractive candidates due to their high power density, fast charge–discharge capability, long cycle life, and compatibility with flexible substrates [125]. Microsupercapacitors can be integrated with photonic sensing and energy harvesting units allow temporary storage of excess energy during periods of high availability and its release in low-energy conditions [126]. The design of such integrated systems requires matching the electrical characteristics of the storage component with the output of the harvesting mechanism, as well as ensuring mechanical compatibility and minimal footprint. Advances in printable and stretchable energy storage materials further support seamless integration into wearable platforms [127].

The relevance of microsupercapacitors for on-device energy storage is further supported by recent advances in flexible and stretchable devices, such as the laser-induced graphene-based microsupercapacitors reported by Han et al. [128]. These devices exhibit a wide operating voltage window up to 1.5 V, high areal capacitance of 2.3 mF cm^−2^, and stable electrochemical performance with more than 93% capacitance retention after 5000 charge–discharge cycles, confirming their suitability for long-term energy buffering in wearable and miniaturized systems. Importantly, the devices are fabricated on polydimethylsiloxane substrates, enabling excellent mechanical flexibility and stretchability while maintaining electrochemical stability under repeated deformation, which is critical for integration with soft and conformal sensing platforms [128].

**Figure 10:**
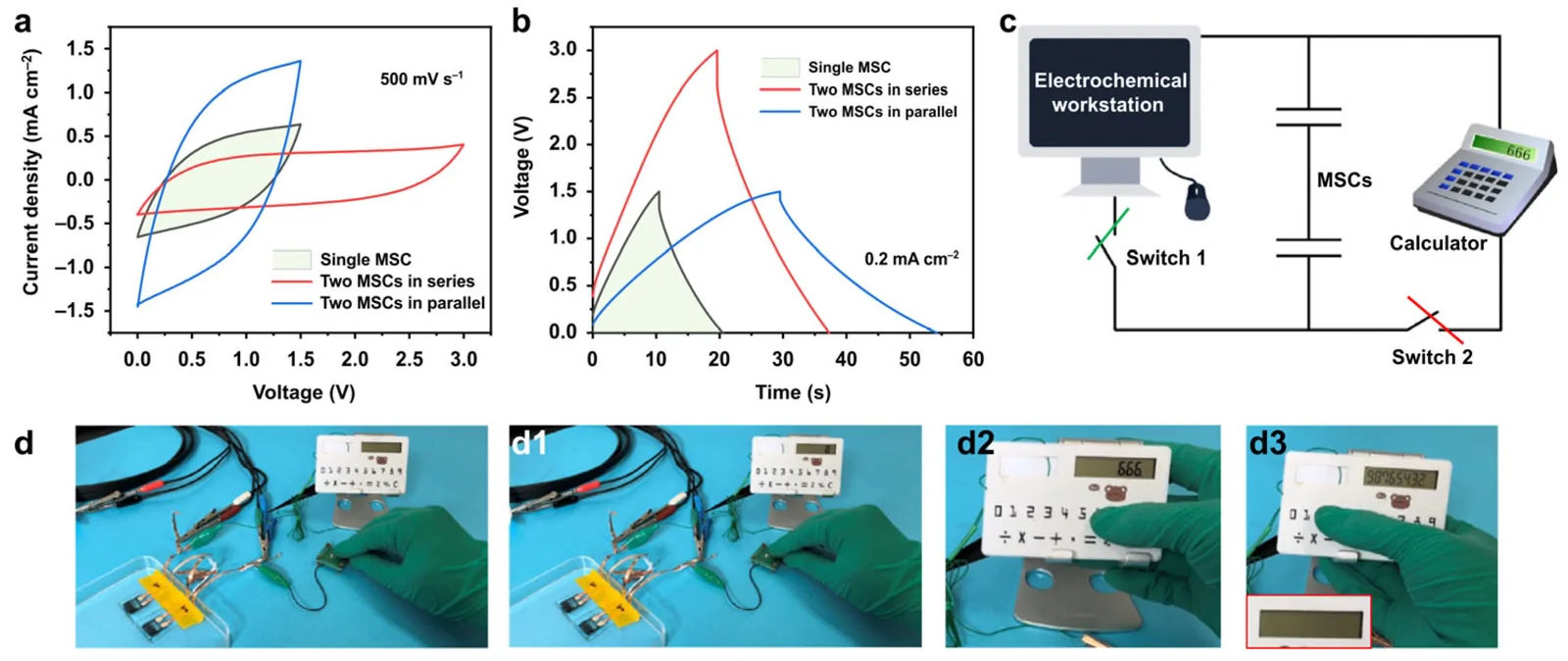
(**a**) Cyclic voltammetry curves recorded at a scan rate of 500 mV s^−1^; (**b**) galvanostatic charge–discharge profiles of the MSC-300 device measured at a current density of 0.2 mA cm^−2^; (**c**) schematic illustration of the equivalent circuit used for the energy storage and delivery system; (**d**) photographic images (**d1**–**d3**) demonstrating the practical application setup of the microsupercapacitor powering an external electronic device [128].

The electrochemical behavior of the microsupercapacitors demonstrates rapid charge–discharge characteristics and high-power delivery capability, as evidenced by nearly ideal capacitive behavior in cyclic voltammetry and symmetric triangular profiles in galvanostatic charge–discharge measurements, indicating efficient ion transport and low internal resistance. Furthermore, the ability to tailor electrode geometry, such as reducing electrode width, leads to enhanced capacitance and improved energy and power density, highlighting the importance of device architecture optimization for matching the electrical output of energy harvesters [128]. A key demonstration of practical energy storage functionality is provided through the application tests illustrated in Figure 10a–d. The cyclic voltammetry curves in Figure 10a show that when two microsupercapacitors are connected in series, the operating voltage window is effectively extended from approximately 1.5 V for a single device to about 3 V, while parallel configuration increases the current response, reflecting enhanced charge storage capacity. The corresponding galvanostatic charge–discharge profiles in Figure 10b confirm these behaviors, where series connection increases the discharge voltage and parallel connection prolongs the discharge time, demonstrating tunable energy and power output depending on circuit configuration. The schematic in Figure 10c presents the integration of the microsupercapacitor unit with an external load through a switching circuit, illustrating a practical configuration for energy storage and delivery. In Figure 10d, the charged microsupercapacitors are shown to power a calculator, where a short charging duration of approximately 20 s enables sustained operation for about 130 s, clearly demonstrating the capability of these devices to store excess energy and release it when needed [128].

These findings directly support the role of microsupercapacitors as effective intermediate energy storage components in integrated systems, where they can buffer fluctuating energy inputs from harvesting units and provide stable power output for sensing and processing tasks. The combination of high power density, rapid charge–discharge response, mechanical compliance, and scalable planar architecture makes such devices highly suitable for seamless integration with photonic sensing and energy harvesting modules, particularly in wearable and miniaturized platforms where space, flexibility, and reliability are critical [128].

### 5.5. Power Management Circuits for Ultra-Low-Light Conditions

Effective power management is critical for operating self-powered photonic sensors under ultra-low-light conditions typical of indoor or covered environments [129]. Power management circuits must be capable of operating at very low input voltages and currents, efficiently converting and regulating harvested energy, and minimizing losses. Techniques such as maximum power point tracking, voltage boosting, and adaptive load management are often required to ensure optimal energy utilization [130,131]. In addition, energy-aware system design strategies, including duty cycling, event-driven sensing, and adaptive sampling, can reduce power consumption and extend operational lifetime. The integration of ultra-low-power electronics with photonic sensing elements is therefore essential for achieving reliable and autonomous operation, particularly in scenarios where energy availability is highly constrained [132].

Building upon these requirements, practical implementations of wearable photovoltaic energy harvesting systems demonstrate how power management circuits can be designed to operate under constrained energy conditions while maintaining system functionality. In this context, a wearable energy harvesting platform integrating flexible solar cells, a rechargeable battery, and dedicated power management integrated circuits has been experimentally validated to support continuous operation of a sensor node under realistic usage scenarios [133]. The system incorporates a highly efficient energy harvesting IC capable of initiating operation with input power levels as low as a few microwatts, thereby enabling functionality even under limited illumination conditions. This highlights the necessity of ultra-low-power circuit design, where both the quiescent consumption of control circuitry and the efficiency of energy transfer stages are carefully optimized to avoid dissipating a significant fraction of the harvested energy.

**Figure 11:**
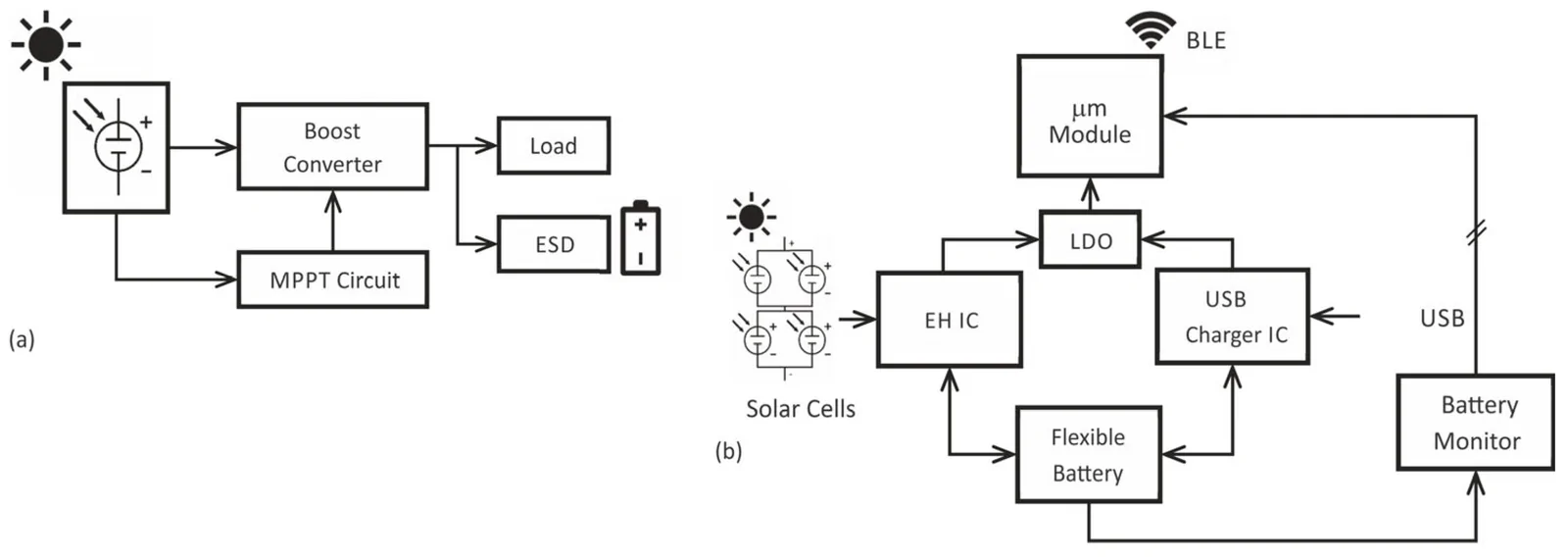
(**a**) Architecture of a photovoltaic energy harvesting circuit with boost conversion and MPPT control, (**b**) schematic of the wearable energy harvesting system with integrated power management, storage, and regulation [133].

A fundamental representation of such power management architectures is illustrated in Figure 11a, where the photovoltaic source is interfaced with a conditioning stage consisting of a boost converter governed by a maximum power point tracking algorithm. This configuration ensures that the solar cells operate near their optimal operating point, typically a fraction of the open-circuit voltage, thereby maximizing the extracted power under varying illumination conditions [133]. The harvested energy is subsequently directed either to an energy storage device or directly to the load, depending on instantaneous power availability. This architecture reflects the need for voltage boosting and adaptive energy routing in ultra-low-light environments, where the raw output of photonic harvesters is often insufficient to directly supply electronic circuits.

In practical wearable implementations, these functional blocks are integrated into compact and highly efficient systems, as depicted in Figure 11b [133]. The system combines a photovoltaic energy harvesting IC with embedded MPPT functionality, a battery charging circuit, and voltage regulation stages to supply a stable output to sensing and communication modules [133]. The energy harvesting IC performs dual roles by both charging the energy storage element and powering the load when sufficient energy is available, while seamlessly transitioning to battery supply during periods of low illumination. A low-dropout regulator is employed to ensure a constant voltage supply to the microcontroller and sensing components, thereby maintaining reliable operation despite fluctuations in harvested energy. Such integration demonstrates the importance of coordinated control between harvesting, storage, and load regulation stages to achieve efficient energy utilization [133]. Experimental results further emphasize the impact of illumination conditions on system performance. Under direct sunlight exposure, the wearable system is capable of sustaining continuous operation provided that sufficient illumination is available for several hours per day, whereas under reduced lighting conditions the harvested energy primarily contributes to extending battery lifetime rather than enabling full autonomy [133]. This behavior underscores the importance of combining efficient power management circuits with energy-aware system strategies, such as adaptive operation and load management, to ensure reliable performance in environments where energy availability is intermittent and highly variable.

To systematically organize the diverse approaches discussed above, it is useful to distinguish between different co-design strategies based on how sensing and energy harvesting functionalities are physically and architecturally coupled. These strategies range from intrinsically self-powered mechanisms, where signal generation and energy conversion originate from the same photophysical process to system-level approaches that incorporate spectral separation, hybrid energy inputs, or intermediate energy storage to stabilize operation. Each strategy reflects a different balance between simplicity, performance optimization, and robustness under variable environmental conditions. Table 3 summarizes these co-design pathways, highlighting their operating principles, advantages, limitations, and relevance for wearable photonic applications.

## 6. Mechanical, Environmental, and Biophysical Constraints

The practical deployment of self-powered wearable photonic sensors is shaped not only by their intrinsic photophysical performance but also by a range of mechanical, environmental, and biophysical constraints that arise from continuous interaction with the human body and its surroundings [145]. Unlike rigid optoelectronic systems, wearable devices must operate reliably under repeated deformation, changing environmental conditions, and varying skin properties [10,15,146]. These factors influence charge transport, optical coupling, and interfacial stability in complex and often nonlinear ways, making device reliability and signal fidelity dependent on a holistic understanding of their combined effects.

Mechanical deformation, including bending, stretching, and compression, can significantly alter the electronic properties of active materials, especially in low-dimensional semiconductors and thin films [147]. Strain can modify the bandgap, carrier mobility, and junction characteristics, directly impacting photocarrier generation and separation. In piezoelectric materials, deformation also induces polarization charges that affect internal electric fields and carrier transport. While controlled strain can be used advantageously, uncontrolled deformation may introduce defects and increase recombination losses [148]. As a result, effective device design must carefully manage strain through strategies such as neutral mechanical plane engineering, stretchable substrates, and mechanically compliant structures [149]. Under bending, a reversible photocurrent reduction can also arise when deflection decreases the illuminated active area; this current loss was ascribed to the less active area under the deflected condition in a flexible UVC photodetector [81].

Over time, repeated mechanical loading can lead to fatigue-related degradation, including microcracking, fracture of brittle components, and interfacial delamination. Cracks disrupt electrical pathways and reduce efficiency, while delamination, often caused by mismatched mechanical properties or weak adhesion, compromises both electrical performance and environmental protection [150,151]. These issues are further intensified by cyclic strain and temperature changes. Mitigating such degradation requires materials with high fracture resistance, strong interfacial bonding, and compatible mechanical properties, along with device architectures that minimize strain in sensitive regions and utilize flexible interconnects.

The optical interface between the device and human skin is another critical factor affecting performance. Human skin is a highly scattering and absorbing medium, with optical behavior influenced by wavelength, hydration, and pigmentation. Light scattering reduces signal intensity and spatial resolution, while air gaps at the interface introduce reflection losses due to refractive index mismatch [152,153]. These limitations are particularly significant under low-light conditions typical of self-powered systems. Improving optical coupling therefore involves using soft, conformal materials that ensure close contact with the skin, as well as incorporating optical matching layers and light-trapping structures to enhance photon utilization [154].

Environmental and physiological factors such as sweat, humidity, and temperature fluctuations also play a major role in device behavior [155,156]. Sweat introduces moisture, ions, and biomolecules that can alter surface conductivity and trigger unwanted electrochemical reactions. Humidity may cause swelling or degradation of polymer components, while temperature changes affect carrier dynamics, thermoelectric behavior, and material stability. Together, these effects can lead to signal drift, increased noise, and long-term degradation. Ensuring reliable operation requires flexible yet effective encapsulation methods that prevent moisture ingress, along with materials that remain stable under physiological conditions.

For practical wearable applications, long-term reliability and washability are essential considerations. Devices must withstand repeated mechanical stress, environmental exposure, and cleaning processes without significant performance loss. Washing introduces additional challenges such as water exposure, detergents, and mechanical agitation, all of which can damage materials and interfaces [157]. Achieving durability therefore depends on chemically stable materials, robust encapsulation, and mechanically resilient interconnects. Maintaining consistent calibration over time is equally important, as aging-related effects like trap formation and electrode degradation can cause signal drift. Emerging solutions, including self-healing materials, stretchable protective layers, and redundant device designs, offer promising approaches to improving durability and enabling real-world use [158,159].

To establish wearable reliability, studies should report a minimum validation set rather than only normalized output after limited bending cycles: bending radius or applied strain, device thickness and neutral-plane position where relevant, cycle number and frequency, and simultaneous optical and electrical measurements during deformation. For textile devices, the washing protocol and post-wash performance should be specified; humidity or artificial-sweat exposure, temperature cycling, adhesion stability, encapsulation-barrier properties, and post-test calibration drift should also be reported. For skin-contact systems, biocompatibility, skin temperature rise, electrical safety, sweat accumulation, skin-tone and body-location dependence, motion artifacts, and inter-subject variation should be evaluated under realistic use conditions [147,148,150,153,159].

## 7. Sensing Modalities and Applications

The diversity of sensing modalities enabled by self-powered wearable photonic systems reflects the versatility of light–matter interactions across physiological, environmental, and technological domains. Unlike conventional wearable sensors that rely on electrical or electrochemical transduction alone, photonic approaches provide access to rich spectral, temporal, and spatial information with minimal invasiveness. When combined with self-powered operation, these systems offer the potential for continuous, maintenance-free monitoring in real-world conditions. However, the practical implementation of such modalities requires careful consideration of the underlying photophysical mechanisms, available energy budget, and the interaction between optical signals and complex biological or environmental media. As a result, application-driven design must remain closely tied to physics-aware optimization to ensure both sensitivity and reliability [160].

### 7.1. Wearable Photoplethysmography (PPG) and Pulse Oximetry

Building on these advances, optical sensing has become a key approach for wearable physiological monitoring due to its ability to provide continuous, noninvasive measurements through light–tissue interaction [161]. Among the available techniques, photoplethysmography has gained prominence because it can capture cardiovascular dynamics using compact, low-power, and skin-compatible devices, making it highly suitable for integration into modern wearable systems. Photoplethysmography is one of the most widely adopted optical sensing techniques in wearable devices, enabling noninvasive monitoring of blood volume changes through light absorption and scattering in tissue [5]. In pulse oximetry, dual-wavelength illumination is used to estimate blood oxygen saturation based on differential absorption of oxygenated and deoxygenated hemoglobin [162]. Self-powered implementations of PPG leverage ambient light or integrated photovoltaic elements to detect pulsatile signals without external bias. The main challenges in this modality include low signal amplitude under weak illumination, strong scattering in skin, and susceptibility to motion artifacts [163]. Achieving reliable operation requires optimizing wavelength selection, detector sensitivity, and optical coupling to the skin, as well as incorporating signal processing strategies to extract physiological information from noisy measurements.

**Figure 12:**
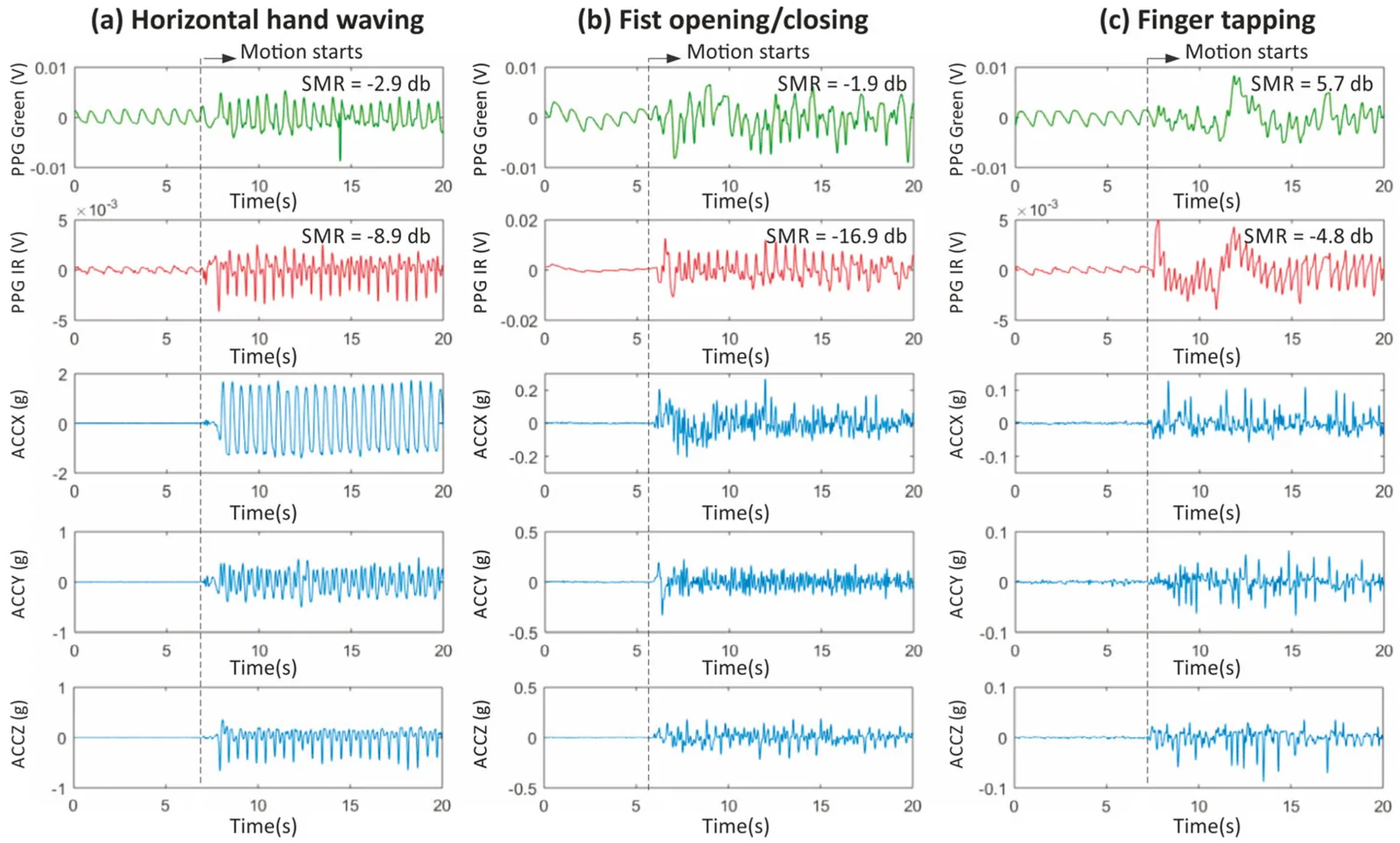
Comparison of green and infrared PPG signals with three-axis acceleration data (X, Y, and Z) under three types of periodic motion: (**a**) horizontal hand waving, (**b**) fist opening and closing, and (**c**) index finger tapping. The *y* axis scales differ across plots due to variations in signal magnitude, and the corresponding signal-to-motion ratio values are indicated within each subplot [164].

The impact of motion artifacts on wearable PPG performance is clearly illustrated in Figure 12a–c [164], where green and infrared PPG signals are compared with three-axis accelerometer data under different motion conditions. During large movements such as hand waving, the accelerometer captures periodic motion that corresponds with distortions in both PPG signals. However, in cases of micromotions such as finger tapping or fist movement, the accelerometer shows minimal variation while the PPG signals are still significantly corrupted, indicating that inertial sensing alone cannot fully characterize motion artifacts. The figure also shows that the infrared PPG signal exhibits stronger motion-related components across all scenarios, while the green PPG signal retains a more pronounced pulsatile waveform associated with blood volume changes. This contrast highlights the dual nature of motion artifacts, arising from both external displacement and internal tissue deformation, and supports the need for wavelength optimization and advanced signal processing techniques to reliably extract physiological information in wearable optical sensing systems [164].

### 7.2. UV Exposure and Dosimetry

Monitoring ultraviolet exposure is important because the biological effects depend on both wavelength and cumulative dose. Terrestrial solar exposure consists predominantly of UVA and a smaller UVB component, whereas UVC monitoring is mainly relevant to artificial sources used for disinfection, sterilization, and industrial processing. Excessive UVA exposure contributes strongly to photoaging and long-term skin damage, while UVB is a major cause of erythema, direct DNA damage, and increased skin-cancer risk. Controlled UVB exposure also contributes to cutaneous vitamin D synthesis, and carefully prescribed UVA or UVB doses are used in phototherapy. Band-resolved and cumulative UV monitoring may therefore support the management of sunburn, photoaging, and exposure associated with carcinogenic risk while also helping control beneficial or therapeutic UV doses [165,166,167,168].

UV monitoring devices can be divided into electrically self-powered photodetectors and passive colorimetric dosimeters. Electrically self-powered photodetectors generate a photocurrent or photovoltage without an externally applied bias and are generally designed to provide a repeatable, time-resolved electrical response, although recovery may be limited by persistent photoconductivity or trapping effects. However, their signal acquisition, processing, display, and wireless communication components may still require electrical power. Passive colorimetric dosimeters instead produce a dose-dependent change in color or optical absorbance. Their output is usually interpreted visually or quantified using a camera or optical reader, and their suitability for repeated use depends on whether the photochemical response is reversible, recoverable, or permanently records the accumulated dose. These devices should therefore be evaluated separately in terms of output type, dose integration, reversibility, readout method, and power requirements.

Recent wearable and flexible systems demonstrate complementary approaches to environmental UV monitoring. Lin et al. developed an autonomous wearable system in which solar-energy harvesting powers separate UVA and UVB measurement channels for continuous monitoring and real-time feedback during daily activities [164]. This represents system-level energy autonomy and should be distinguished from zero-bias operation of the photodetector itself [166]. Singh et al. reported a flexible, paper-based UV sensor employing an indium–gallium–zinc-oxide sensing layer, with wireless transmission demonstrated from devices attached to skin and clothing [169]. Such electronic platforms enable continuous irradiance measurements, whereas spectrally selective colorimetric dosimeters can provide a direct cumulative-dose indication without continuous electronic operation. Accurate healthcare interpretation nevertheless requires band-specific calibration because UVA, UVB, and UVC differ substantially in environmental occurrence and biological effect.

A representative example is the spectrally selective colorimetric dosimetry system based on a photoelectrochromic phosphomolybdic acid–lactic acid platform, which enables differentiation between UVA, UVB, and UVC radiation through distinct optical responses [170]. The system exploits the multiple redox states of the active material, enabling wavelength-dependent reduction processes that produce measurable and visually distinguishable color changes correlated with both radiation type and cumulative dose. The passive dosimeter requires no electrical power for UV sensing and provides a cumulative optical output that can be interpreted directly by the naked eye or quantified through reflectance measurements or camera-based imaging. The reported paper device is intended as a low-cost, disposable indicator, and no rapid color-reset or recovery procedure was demonstrated; it should therefore be distinguished from reversible electrical photodetectors used for continuous irradiance measurements. Nevertheless, it demonstrates high sensitivity to low ultraviolet doses relevant to typical solar exposure conditions and maintains robust performance under variations in temperature and humidity and in the presence of visible and infrared radiation. Furthermore, the platform can be calibrated according to minimal erythemal dose values, enabling personalization for different skin phototypes and supporting its application in wearable ultraviolet monitoring [170].

**Figure 13:**
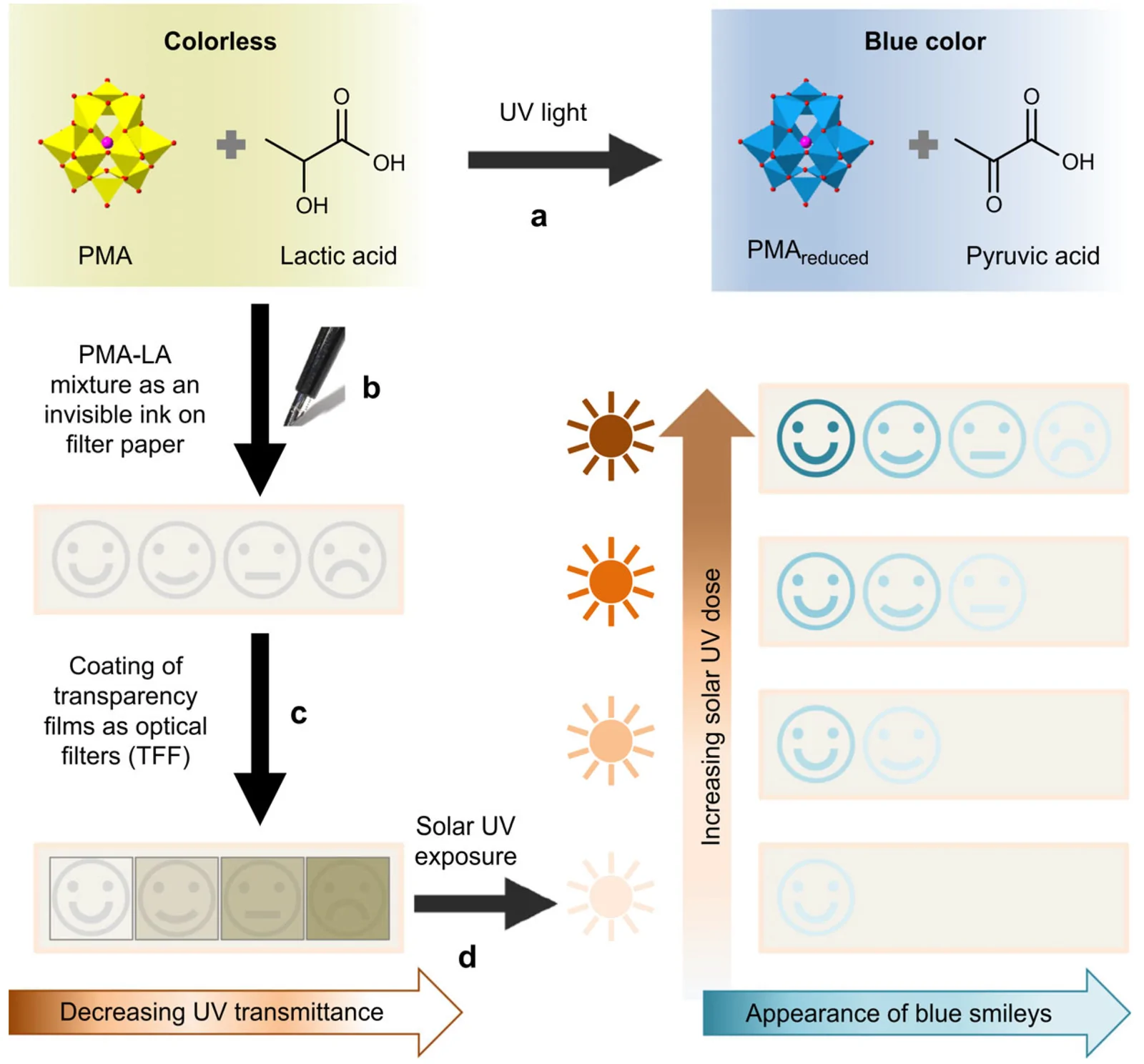
Schematic representation of a colorimetric paper-based ultraviolet sensor. (**a**) An aqueous photoelectrochromic system composed of phosphomolybdic acid and lactic acid undergoes ultraviolet-induced reduction, leading to the formation of a blue-colored product. (**b**) This mixture is utilized as an invisible ink to create patterned indicators on a filter paper substrate. (**c**) The patterned regions are subsequently covered with varying numbers of transparency film layers, which progressively decrease ultraviolet transmittance and enable controlled modulation of the response time across the sensor. (**d**) Upon exposure to solar ultraviolet radiation, the initially invisible patterns gradually develop blue coloration from left to right, corresponding to increasing cumulative ultraviolet dose. The sequential appearance of one to four indicators reflects approximately 25, 50, 75, and 100% of the safe exposure threshold, respectively, while variation in the number of filter layers allows customization of the sensor for different skin phototypes [170].

The working principle and device configuration, as illustrated in Figure 13a–d, demonstrate the progression from material response to practical dosimetry. Initially, phosphomolybdic acid remains nearly colorless but undergoes ultraviolet-induced reduction in the presence of an electron donor, forming a blue-colored reduced species that serves as the sensing signal [170]. This photoresponsive system can then be formulated as an invisible ink and deposited onto flexible substrates such as paper, remaining optically inactive prior to irradiation. To enable controlled and dose-dependent response behavior, optical filter layers are introduced to regulate the amount of ultraviolet radiation reaching different regions of the sensor. Upon exposure to solar ultraviolet radiation, the previously invisible patterns progressively develop visible coloration with increasing intensity, corresponding to cumulative exposure levels, thereby allowing direct visual assessment of ultraviolet dose without the need for electronic readout [170].

### 7.3. Chemical and Biochemical Photonic Sensing

Photonic techniques enable selective detection of chemical and biochemical species through mechanisms such as optical absorption, fluorescence, and refractive index modulation [171,172,173]. In wearable systems, these approaches can be used to monitor analytes in sweat, interstitial fluid, or the surrounding environment. Self-powered operation can be achieved through photogenerated signals in sensing layers or through coupling with energy harvesting components [174]. For example, colorimetric or fluorescence-based sensors can be read by integrated photodetectors powered by ambient light. Challenges in this domain include achieving high specificity in complex biological matrices, maintaining stability of sensing elements under varying pH and humidity, and ensuring consistent optical readout under changing illumination conditions. Integration of selective recognition elements with stable photonic transducers is critical for reliable performance [175].

**Figure 14:**
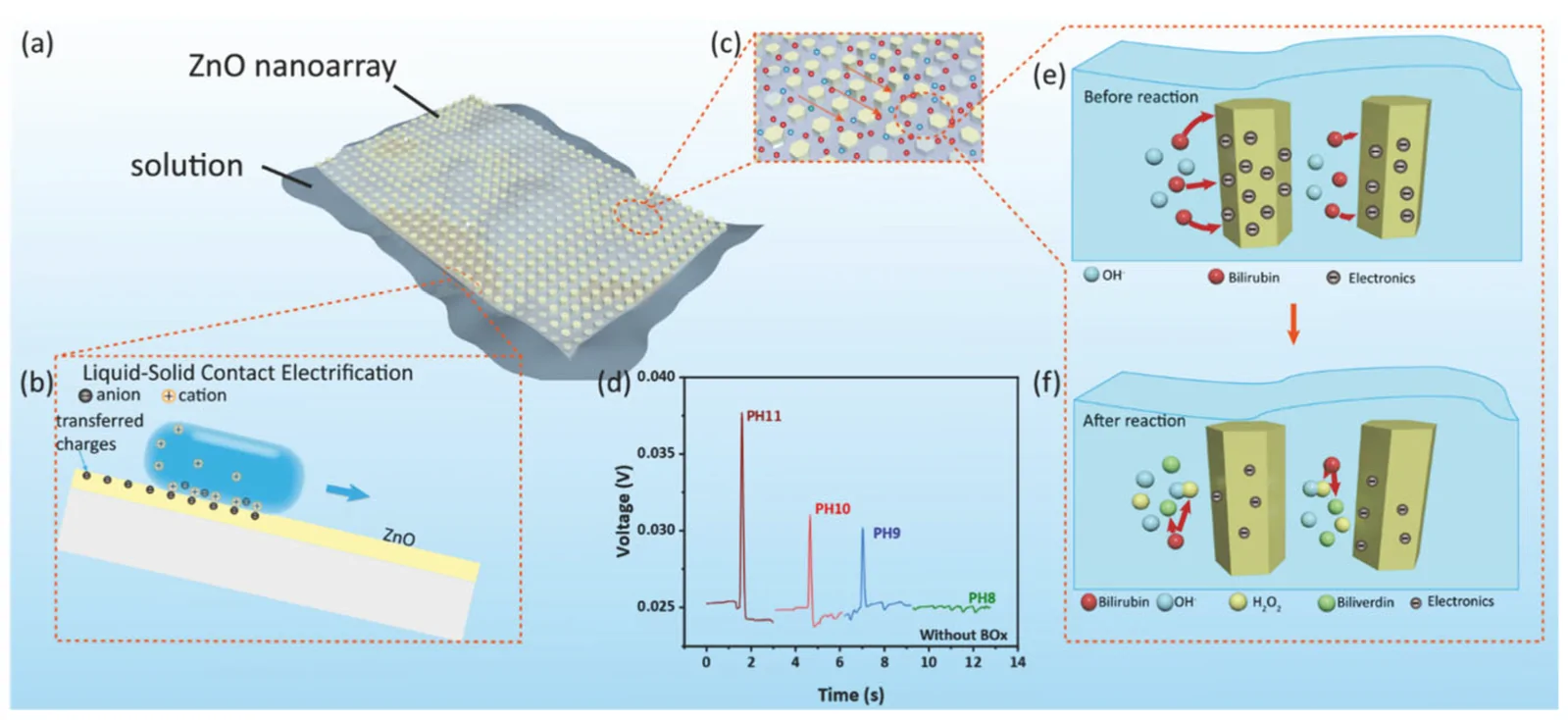
Schematic illustration of the operating mechanism of the self-powered wearable hydrovoltaic biosensor. (**a**,**b**) Generation of electrical output through liquid–solid contact electrification. (**c**) Molecular and ionic interactions during fluid flow along the ZnO nanowire surface. (**d**) Variation in output voltage under different pH conditions, demonstrating environmental sensitivity. (**e**,**f**) Enzymatic biosensing mechanism for bilirubin detection before and after reaction [171].

The following hydrovoltaic example is included as a self-powered biochemical/electrical sensing platform, rather than as a purely photonic sensor, because its analytical signal arises from liquid–solid interfacial charge generation rather than an optical transduction mechanism. A representative example is a self-powered wearable biosensor designed for real-time monitoring of neonatal jaundice through bilirubin detection in urine [171]. The device is embedded within a diaper and operates without an external power source by utilizing a hydrovoltaic effect generated when liquid flows across a ZnO nanoarray. In this system, the generated electrical output simultaneously functions as both the energy source and sensing signal. The biochemical selectivity is achieved through the immobilization of bilirubin oxidase, which catalyzes reactions that modulate the electrical output depending on analyte concentration, demonstrating reliable sensitivity, specificity, and repeatability for physiological monitoring [171]. The working mechanism of this device is illustrated in Figure 14a–f, where both energy generation and biochemical sensing processes are integrated. In Figure 14a,b, the fundamental self-powered operation is shown through the contact electrification process at the liquid–solid interface. When urine flows across the ZnO nanoarray surface, charge transfer occurs due to interactions between the liquid and the nanostructured material, resulting in the generation of an electrical potential. This hydrovoltaic effect enables the device to function without any external power supply, as the mechanical motion of the liquid itself drives signal generation [171].

Figure 14c further illustrates the molecular-level interaction of the liquid flowing along the ZnO nanowires, where ions and molecules in the solution influence the distribution and movement of charges at the interface. This interaction directly affects the magnitude of the generated electrical signal. The influence of the chemical environment is experimentally validated in Figure 14d, where variations in pH lead to measurable changes in output voltage, confirming that ionic concentration and chemical conditions modulate the hydrovoltaic response. This demonstrates that the sensing platform is inherently sensitive to changes in the surrounding biochemical environment. The biochemical sensing mechanism is depicted in Figure 14e,f, which show the system before and after the enzymatic reaction. Prior to the reaction, bilirubin molecules and ions are distributed near the ZnO surface without significantly altering the charge transfer process. After the introduction of bilirubin oxidase, an enzymatic reaction converts bilirubin into biliverdin and hydrogen peroxide, followed by further oxidation that releases protons and electrons. These reaction products alter the ionic balance and interfacial charge distribution, thereby modulating the contact electrification process and changing the output voltage. As a result, the electrical signal becomes directly correlated with bilirubin concentration, enabling quantitative biochemical sensing. This coupling of enzymatic activity with hydrovoltaic signal generation represents a key strategy for achieving self-powered, wearable biosensing systems [171].

### 7.4. Environmental Light and Radiation Monitoring

Self-powered wearable photonic sensors are well suited for monitoring environmental light intensity, spectral composition, and radiation exposure. Such capabilities are relevant for applications ranging from circadian rhythm regulation to occupational safety in high-radiation environments [166]. Devices can be designed to respond selectively to specific wavelength ranges, enabling differentiation between visible light, infrared radiation, and ionizing or nonionizing radiation. The use of photovoltaic or photothermoelectric mechanisms allows these sensors to operate continuously without external power. However, accurate environmental monitoring requires calibration against varying ambient conditions, as well as compensation for temperature and angle-dependent effects [176]. Long-term stability and resistance to environmental degradation are also essential for reliable operation. In this context, photothermoelectric sensing platforms have emerged as effective solutions for self-powered environmental light and radiation monitoring, offering simple device architectures and reliable signal generation based on thermal gradients induced by incident radiation [177].

In this regard, a flexible photothermoelectric sensor based on Bi_2_Te_3_ and Sb_2_Te_3_ thin films has been demonstrated for self-powered optical power detection, utilizing the conversion of absorbed light into heat and subsequent thermoelectric voltage generation [178]. The device exhibits a strong linear relationship between photovoltage and incident optical power, with high accuracy and minimal error, enabling reliable quantification of radiation intensity. It also shows rapid response and recovery behavior, along with stable and repeatable performance under cyclic illumination conditions. The radial device architecture enhances thermal gradients, thereby improving sensitivity, while the absence of external power requirements supports continuous and autonomous operation. Furthermore, the ability to define threshold voltages corresponding to specific radiation levels enables practical implementation in safety monitoring systems [178].

**Figure 15:**
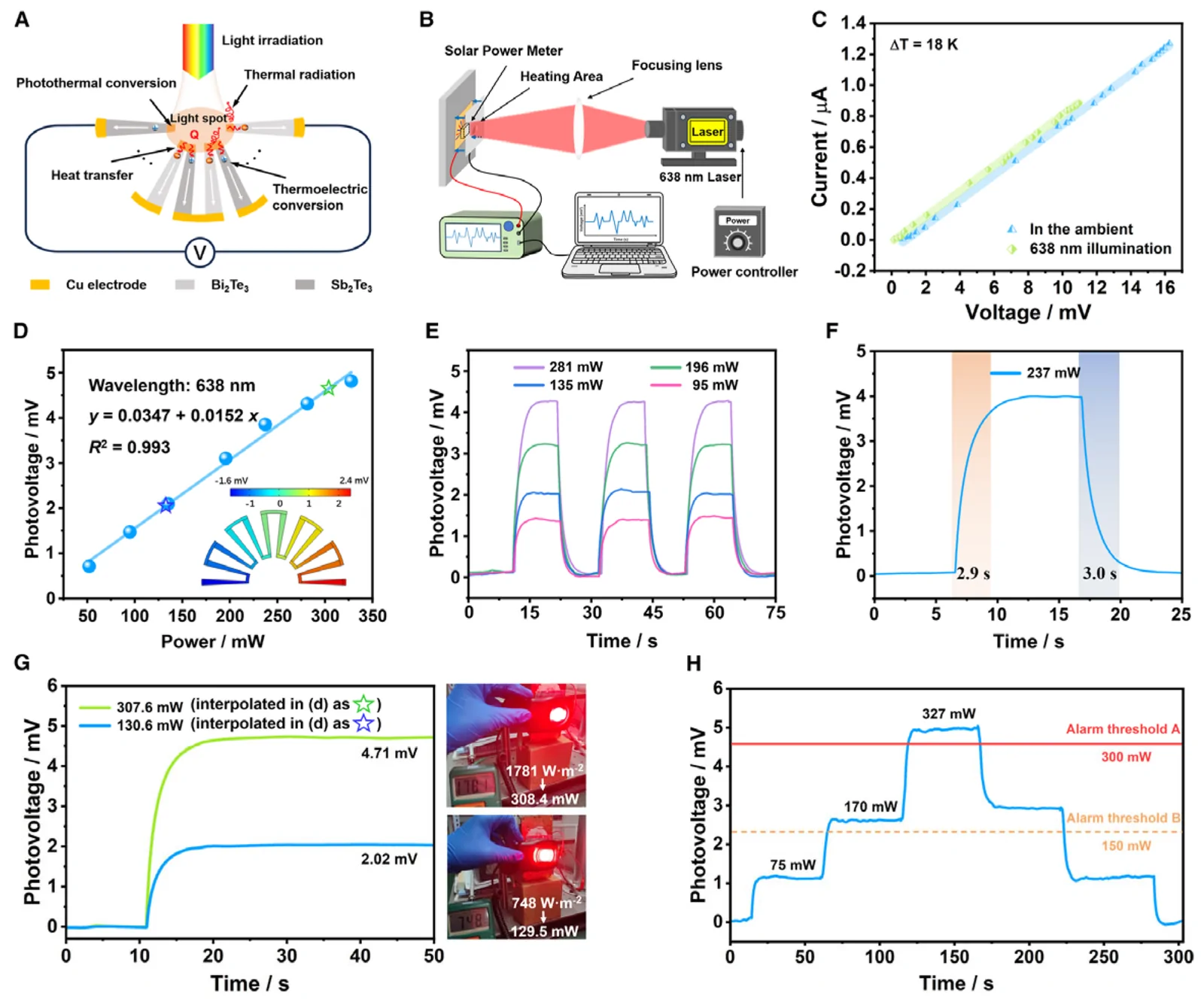
Photothermoelectric characterization of the thermoelectric sensor under 638 nm laser irradiation. (**A**) Schematic of photothermal and thermoelectric conversion mechanism. (**B**) Experimental setup for laser sensing. (**C**) Current voltage characteristics under illumination. (**D**) Linear dependence of photovoltage on incident power with spatial voltage mapping inset. (**E**) Transient response under varying power. (**F**) Response and recovery behavior at 237 mW. (**G**) Comparison of calculated and measured optical power from photovoltage. (**H**) Photovoltage-based threshold detection for radiation monitoring [178].

The sensing mechanism and performance characteristics are illustrated in Figure 15A–H. The device operates by absorbing incident radiation at the electrode surface, generating a localized temperature gradient that drives charge carrier diffusion through the Seebeck effect, producing a measurable photovoltage. Experimental validation using a controlled laser source confirms the capability of the sensor to detect optical power under defined conditions. The photovoltage exhibits a linear dependence on incident radiation intensity, supporting accurate calibration for environmental monitoring applications. Transient measurements demonstrate fast and stable response behavior, while comparison between calculated and measured optical power values indicates high sensing accuracy. The implementation of threshold-based voltage levels further enables classification of radiation exposure into different regimes, highlighting the potential of the device for real-time environmental monitoring and wearable safety applications [178].

## 8. Manufacturing, Scalability, and Sustainability

The translation of self-powered wearable photonic sensors from laboratory prototypes to widespread real-world deployment depends critically on manufacturing scalability, material sustainability, and system-level reliability. While many high-performance devices have been demonstrated under controlled conditions, their fabrication often relies on processes that are not readily compatible with large-area, low-cost, or high-throughput production [179]. At the same time, wearable applications impose additional constraints related to biocompatibility, environmental exposure, and long-term durability. Therefore, scalable manufacturing approaches must be developed in parallel with sustainable material choices and robust packaging strategies, while also aligning with regulatory and clinical requirements. Addressing these factors holistically is essential for enabling practical adoption across healthcare, consumer electronics, and environmental monitoring domains.

### 8.1. Roll-to-Roll and Printing-Compatible Processes

Scalable fabrication of wearable photonic devices increasingly relies on solution-based and printing-compatible techniques that enable continuous, high-throughput production on flexible substrates [8]. Roll-to-roll processing, inkjet printing, screen printing, gravure printing, and aerosol jet deposition offer pathways for depositing semiconducting layers, electrodes, and encapsulation materials over large areas with reduced cost and material waste [180,181,182,183]. These methods are particularly well suited for organic semiconductors, nanomaterials, and hybrid composites that can be formulated into printable inks [184]. However, achieving uniform film morphology, controlled thickness, and reproducible electrical performance across large areas remains a significant challenge. Variations in solvent evaporation, ink rheology, and substrate interactions can lead to defects, inhomogeneity, and performance variability. Furthermore, integrating multiple functional layers with different processing requirements into a single continuous manufacturing line requires careful optimization of process compatibility and thermal budgets [185].

In this context, recent advances in fully printable optoelectronic devices provide important insights into both the opportunities and limitations of such fabrication strategies. The work reported by Bairaktaris et al. demonstrates a scalable approach for the realization of flexible photodetectors using solution-processed materials and printing-compatible techniques [186]. The devices are fabricated using a simplified architecture that relies on additive deposition of functional layers on flexible substrates, highlighting the potential of low-temperature and low-complexity processes for large-area manufacturing. The compatibility of the materials with inkjet printing and other solution-based methods further underscores their suitability for integration into roll-to-roll production schemes.

A key outcome of this study is the demonstration that relatively simple, printable device structures can deliver sufficient performance for practical sensing applications, even in the presence of material and process limitations. Organic semiconductor-based photodetectors exhibit measurable and stable photoresponse with on–off ratios adequate for distinguishing illumination conditions, while maintaining mechanical flexibility under repeated bending. This reinforces the viability of printable materials for wearable photonic systems, where conformability and mechanical robustness are essential. At the same time, the study highlights that system-level design considerations can partially compensate for device-level variability, for instance through the use of reference sensing schemes and tolerant signal processing architectures [186].

**Figure 16:**
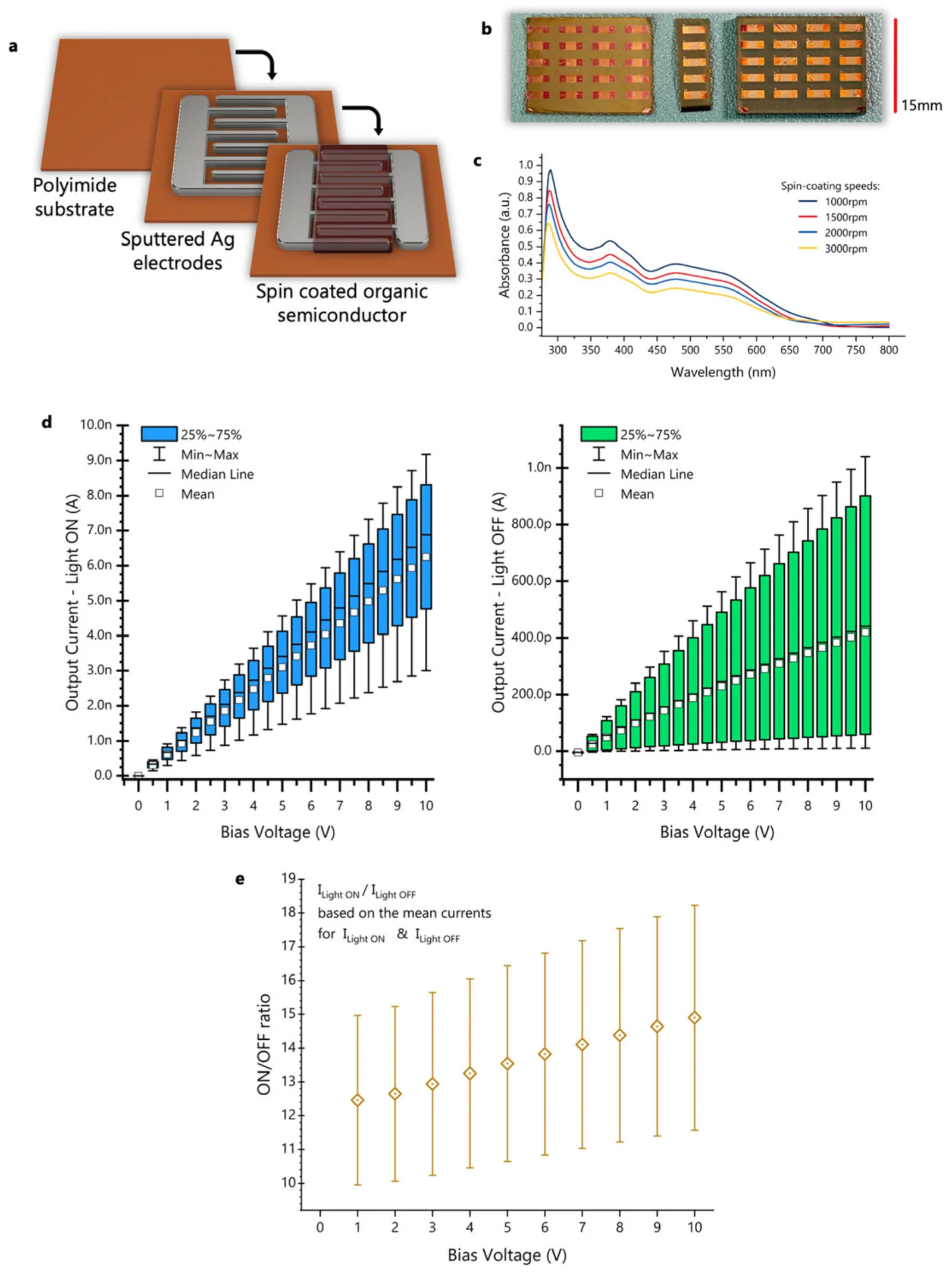
(**a**) Fabrication process of flexible photodetectors with spin-coated organic semiconductor on polyimide substrate, (**b**) photographs of device arrays on polyimide before and after cutting and control devices on glass, (**c**) absorption spectra of the organic semiconductor at different film thicknesses, (**d**) statistical distribution of light and dark currents for 20 devices on polyimide, (**e**) on–off ratio as a function of bias voltage with error bars indicating [186].

Importantly, the results also provide direct experimental evidence of the challenges associated with printing-compatible fabrication. Significant variability in electrical characteristics is observed across nominally identical devices, primarily attributed to nonuniform film formation during solution deposition. Variations in semiconductor thickness, which arise from differences in solvent evaporation dynamics and coating conditions, lead to a broad distribution of current levels and on–off ratios. This behavior is consistent with the broader limitations of printed electronics, where precise control over morphology and thickness remains difficult to achieve across large areas. Such variability poses a critical barrier to reproducibility and device reliability, particularly when scaling from laboratory demonstrations to industrial manufacturing [186]. These effects are clearly illustrated in Figure 16 of the study. The fabrication sequence shown in Figure 16a demonstrates a minimal processing route involving deposition of electrodes followed by solution coating of the active semiconductor layer on a flexible polyimide substrate, emphasizing compatibility with scalable and potentially roll-to-roll processes. The corresponding device images in Figure 16b confirm the successful realization of arrays on both flexible and rigid substrates, highlighting the versatility of the approach. The optical absorption characteristics presented in Figure 16c reveal strong coverage across the visible spectrum, which is essential for ambient light sensing and illustrates the advantage of organic semiconductors over oxide counterparts in this application.

The statistical analysis in Figure 16d provides a clear indication of device-to-device variability, with a wide spread in both light and dark current values across multiple samples. This dispersion directly reflects inconsistencies in film thickness and morphology inherent to solution processing on flexible substrates. Figure 16e further shows the dependence of the on–off ratio on applied bias, indicating that while performance can be tuned electrically, variability persists across the device population. Together, these observations underscore the central challenge identified in printing-based fabrication, namely the trade-off between scalability and uniformity. While solution-based techniques enable low-cost, high-throughput production, achieving consistent material deposition and reproducible device performance remains a key obstacle that must be addressed for the widespread adoption of roll-to-roll manufactured wearable photonic systems [186].

### 8.2. Material Availability and Environmental Impact

The choice of materials for wearable photonic systems has important implications for both scalability and sustainability [187]. Some high-performance optoelectronic materials rely on scarce, toxic, or environmentally hazardous elements, which may limit their large-scale adoption [188]. For example, certain perovskite compositions contain lead, raising concerns about environmental safety and long-term disposal. Similarly, the use of rare metals in electrodes or nanostructures can increase cost and supply chain vulnerability. Developing alternative materials that are abundant, non-toxic, and recyclable is therefore an important research direction [189]. In addition, the environmental impact of fabrication processes, including solvent use, energy consumption, and waste generation, must be considered. Sustainable manufacturing approaches emphasize green chemistry, low-temperature processing, and the use of biodegradable or recyclable substrates to reduce the overall ecological footprint [190].

Lead-free CsSn_0·5_Ge_0·5_I_3_ illustrates both the potential and the limitations of replacing Pb-based perovskites. Chen et al. reported a power conversion efficiency of 7.11% and less than 10% efficiency loss after 500 h of continuous operation under one-sun illumination in nitrogen, supported by the spontaneous formation of an ultrathin native oxide passivation layer [188]. This composition eliminates lead from the absorber rather than partially substituting it. However, the reported efficiency and stability do not establish performance equivalence to Pb-based systems, and testing in nitrogen does not demonstrate durability under moisture exposure, sweat, or repeated deformation. Furthermore, solar-cell efficiency alone cannot establish suitability for direct photodetection, which requires evaluation of zero-bias responsivity, noise, response time, and operational drift. The available evidence therefore supports further investigation of CsSn_0·5_Ge_0·5_I_3_ rather than its general replacement of Pb-based materials. Such replacement would require application-specific comparisons under matched operating conditions, demonstrating adequate sensing performance or harvested power alongside durability and safety. Although eliminating lead addresses a specific material hazard, it does not independently establish biocompatibility or the safety of degradation products.

**Figure 17:**
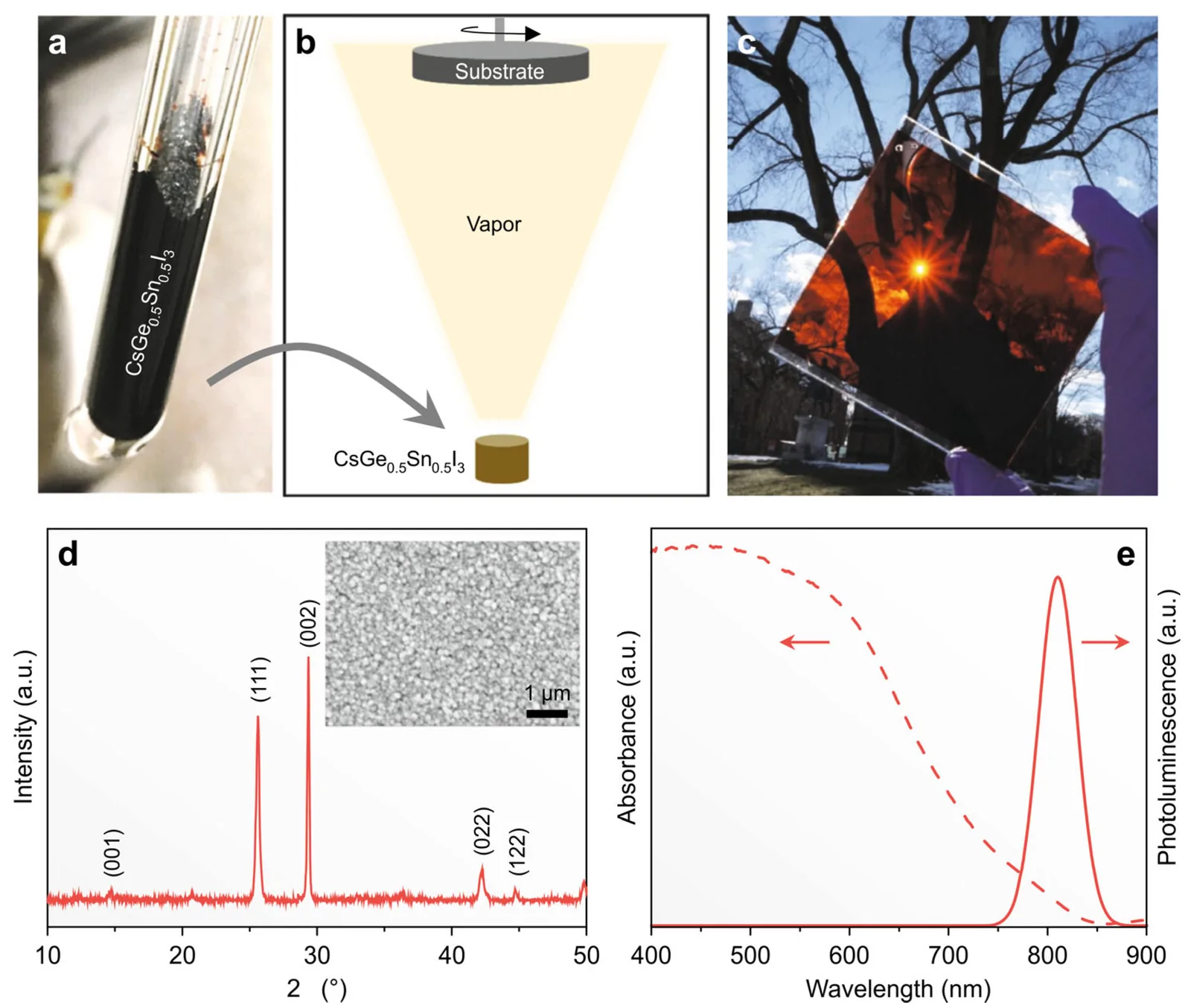
Film synthesis and characterization of CsSn_0·5_Ge_0·5_I_3_ perovskites. (**a**) Photograph of the as-prepared CsSn_0·5_Ge_0·5_I_3_ solid obtained via melt crystallization, (**b**) schematic representation of the single-source evaporation process used for depositing uniform thin films, (**c**) photograph of a large-area CsSn_0·5_Ge_0·5_I_3_ thin film on a glass substrate exhibiting a dark reddish appearance, (**d**) X-ray diffraction pattern confirming the crystalline phase of the deposited thin film, with the inset showing a top view SEM image of the film morphology, (**e**) optical absorption and steady-state photoluminescence spectra of the CsSn_0·5_Ge_0·5_I_3_ thin film [188].

Figure 17 summarizes the synthesis and characterization of CsSn_0·5_Ge_0·5_I_3_ [188]. The photographs show the material obtained by melt crystallization and the deposited film, while the schematic illustrates the single-source evaporation process (Figure 17a–c). The X-ray diffraction pattern supports identification of the crystalline phase, and the electron microscopy image provides evidence of the film morphology (Figure 17d). Optical absorption and photoluminescence measurements characterize its spectral properties (Figure 17e) [188]. These results support the feasibility of preparing photoactive films, but do not independently demonstrate manufacturing scalability, environmental safety, or competitive performance in wearable photonic devices.

### 8.3. Encapsulation and Packaging Strategies

Encapsulation and packaging play a crucial role in protecting wearable photonic devices from environmental degradation while maintaining mechanical flexibility and optical functionality [191]. Effective encapsulation must prevent ingress of moisture, oxygen, and contaminants that can degrade active materials, particularly in organic and hybrid systems [192]. At the same time, encapsulation layers must be soft, stretchable, and optically transparent to preserve device performance and user comfort [193]. Multilayer barrier films, elastomeric coatings, and hybrid organic–inorganic encapsulation strategies are commonly employed to achieve these requirements. Packaging design must also account for mechanical robustness, ensuring that devices can withstand repeated bending, stretching, and washing without delamination or failure. Balancing barrier performance with flexibility and breathability remains a key challenge, especially for skin-mounted applications where user comfort is critical [194,195].

Although this highly stretchable conjugated-polymer strain sensor is not a photonic device, it is included as a contextual example because its encapsulation and interfacial-design principles are transferable to wearable photonic systems [191]. In this device, styrene–ethylene–butylene–styrene was used as both the substrate and the encapsulation layer because of its high fracture strain of about 800%, allowing the package to deform with the active material without compromising structural integrity. The encapsulation layer was designed to fully cover the electrode region while preserving electrical connection through patterned vias, thereby reducing exposure of vulnerable interfaces to humid environments. This strategy is particularly relevant to wearable photonic and optoelectronic systems, where interfacial degradation and environmental ingress can rapidly limit long-term performance. The same study also showed that the active sensing film retained high optical transparency, with a visible transmittance of 98.7%, while the complete device remained extremely lightweight at about 45 mg, demonstrating how encapsulation can be integrated without sacrificing functional or mechanical compliance [191].

**Figure 18:**
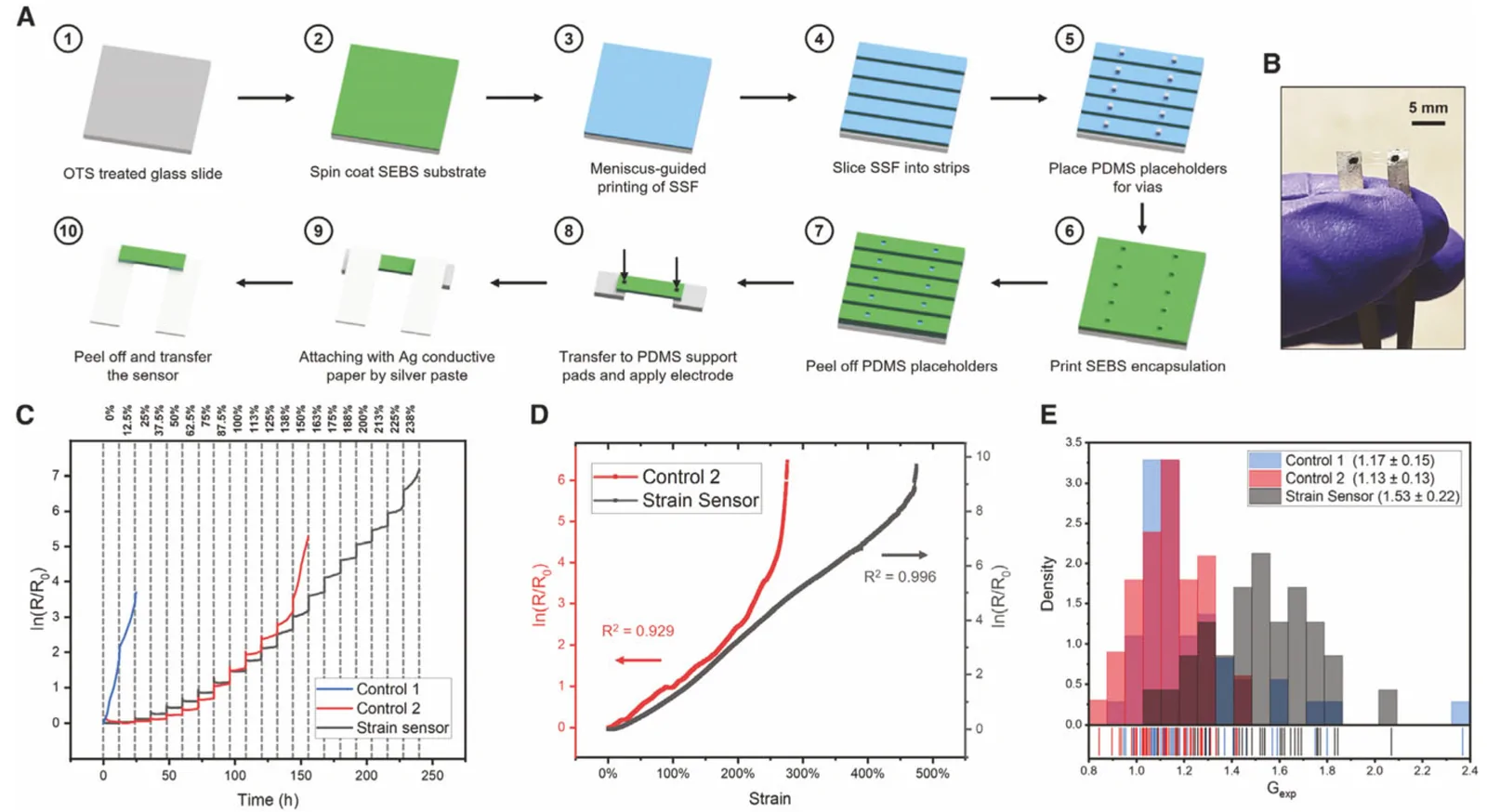
(**A**) Schematic illustration of the strain sensor fabrication process, (**B**) optical image of the fully fabricated and encapsulated device, (**C**) assessment of environmental stability using a step strain protocol, where samples were maintained at 20 °C under 30–50% relative humidity and incrementally stretched by 0.5 mm, corresponding to 12.5% strain for a 4 mm long device, at 12 h intervals, while resistance was continuously recorded at 1 or 5 min intervals, (**D**) comparison of the electromechanical response between Control 2 and the optimized strain sensor under a low strain rate of 0.01 mm/min, demonstrating improved linearity in the encapsulated device, (**E**) statistical distribution of the sensitivity exponent (G_exp_) illustrating device reproducibility, with comparison between the optimized sensor and control samples [191].

The fabrication sequence shown in Figure 18A,B demonstrates a placeholder-assisted encapsulation process that is especially instructive for packaging design. After formation of the strain sensing film, the film was cut into strips approximately 1.5 mm in width, and two PDMS placeholders of 1 × 1 mm were introduced at the two ends before deposition of the SEBS encapsulation layer. After encapsulation, removal of the PDMS placeholders created vias for electrical interconnection, enabling full protection of the active region while maintaining electrode access. The encapsulated strips were then transferred onto two PDMS support pads separated by a 4 mm gap, and conductive contacts were formed using a PEDOT:PSS and single-walled carbon nanotube composite electrode followed by attachment of silver conductive paper. This process produced an encapsulated device architecture that combines environmental protection, stretchability, and reliable electrical integration, and Figure 18B visually confirms the compact and mechanically compliant final sensor geometry [191].

The quantitative results in Figure 18C provide direct evidence for the effectiveness of encapsulation in suppressing environmental degradation. Under a step stretch stability test performed at 30% to 50% relative humidity and 20 °C, with strain increased by 12.5% every 12 h for a 4 mm long device, the unencapsulated control exhibited a degradation rate of approximately 0.11 h^−1^. When encapsulation was introduced using the same silver electrode configuration, the degradation rate decreased to about 0.006 h^−1^, corresponding to an improvement of more than two orders of magnitude. Upon further optimization of the electrode interface through the PEDOT:PSS and carbon nanotube composite electrode, the degradation rate decreased again to 0.0008 h^−1^. These data show that encapsulation alone provides a substantial barrier effect, but the highest stability is obtained when barrier design is combined with improved interfacial adhesion and complete sealing of the electrode region [191].

Figure 18D further shows that encapsulation and interface engineering affect not only environmental stability but also electromechanical fidelity. The encapsulated control device with a conventional silver electrode displayed a nonlinear response with an R^2^ value of 0.929 and a strain sensing range below 300%. In contrast, the fully engineered encapsulated sensor with the composite electrode exhibited a nearly ideal linear response with an R^2^ of 0.996 and a sensing range of 500%. The improvement was attributed to reduced stiffness mismatch between the soft sensing film and the electrode layer, which suppressed interfacial electrical failure at high strain. For wearable photonic systems, this result is highly relevant because packaging layers must not only isolate devices from the environment but also preserve signal consistency during repeated bending and stretching [191].

Figure 18E addresses reproducibility, another critical consideration in packaging and encapsulation design. The sensitivity exponent of the fully encapsulated sensor was reported as 1.53 ± 0.22, compared with 1.17 ± 0.15 for the unencapsulated control and 1.13 ± 0.13 for the encapsulated silver electrode control. Although the fully engineered device showed a somewhat broader distribution, its coefficient of variation remained only 14.4%, compared with 12.8% and 11.5% for the two controls, indicating that the encapsulation and electrode modifications improved functionality without causing unacceptable variability. Taken together, Figure 18A–E demonstrates that successful packaging of wearable devices requires more than a passive barrier layer. Rather, optimal performance emerges from a coordinated strategy involving soft encapsulation materials, complete coverage of critical interfaces, patterned electrical access, and interfacial mechanical matching. Such principles are directly applicable to wearable photonic devices, where stable optical and electronic performance must be maintained under complex environmental and mechanical loading conditions [191].

### 8.4. Life Cycle Analysis

A comprehensive evaluation of wearable photonic systems must include life cycle analysis that considers the environmental and economic impacts from material extraction and device fabrication to usage, maintenance, and end-of-life disposal. Life cycle assessment frameworks can help identify key stages where energy consumption, material waste, or environmental harm are most significant, enabling targeted improvements [196,197,198]. For example, reducing reliance on hazardous materials, improving device longevity, and enabling recycling or safe disposal can substantially lower environmental impact. In wearable applications, considerations such as washability, repairability, and reusability also influence the overall life cycle footprint. Incorporating life cycle thinking into the design process encourages the development of devices that are not only high performing but also sustainable and economically viable in the long term [199].

Recent advances in wearable sensing technologies demonstrate how life cycle assessment can be effectively integrated into the design and optimization of device systems. In a representative study on a wearable microfluidic sweat sensor, life cycle assessment was applied at an early stage to evaluate the environmental impact of materials, fabrication processes, and device architecture [196]. The results reveal that the production phase is the dominant contributor to environmental burden, with printed electronic components, particularly electrodes, accounting for approximately 60 to 90% of the total impact across multiple categories. Among the materials examined, silver-based electrodes exhibit the highest environmental footprint due to energy-intensive extraction and processing, whereas alternative materials such as copper laminates and screen-printed graphite significantly reduce emissions, resource consumption, and toxicity while maintaining functional performance. These findings highlight the critical importance of material selection in reducing the environmental impact of wearable sensor systems [196].

Beyond material considerations, the study demonstrates that life cycle assessment can serve as a powerful design tool for identifying environmental hotspots and guiding sustainable innovation. By incorporating LCA during the development stage, it becomes possible to optimize fabrication processes, reduce energy-intensive steps, and minimize material waste before large-scale production. The analysis also shows that manufacturing techniques such as printing, curing, and laser processing contribute significantly to the overall footprint, indicating that process simplification and efficiency improvements are essential for sustainable device fabrication. In addition, the device adopts a hybrid architecture consisting of a reusable electronic module coupled with a disposable microfluidic cartridge, which reduces overall material consumption and supports more sustainable usage patterns. Considerations related to recyclability, disassembly, and material recovery are also emphasized, reinforcing the need for circular design strategies in wearable systems [196].

**Figure 19:**
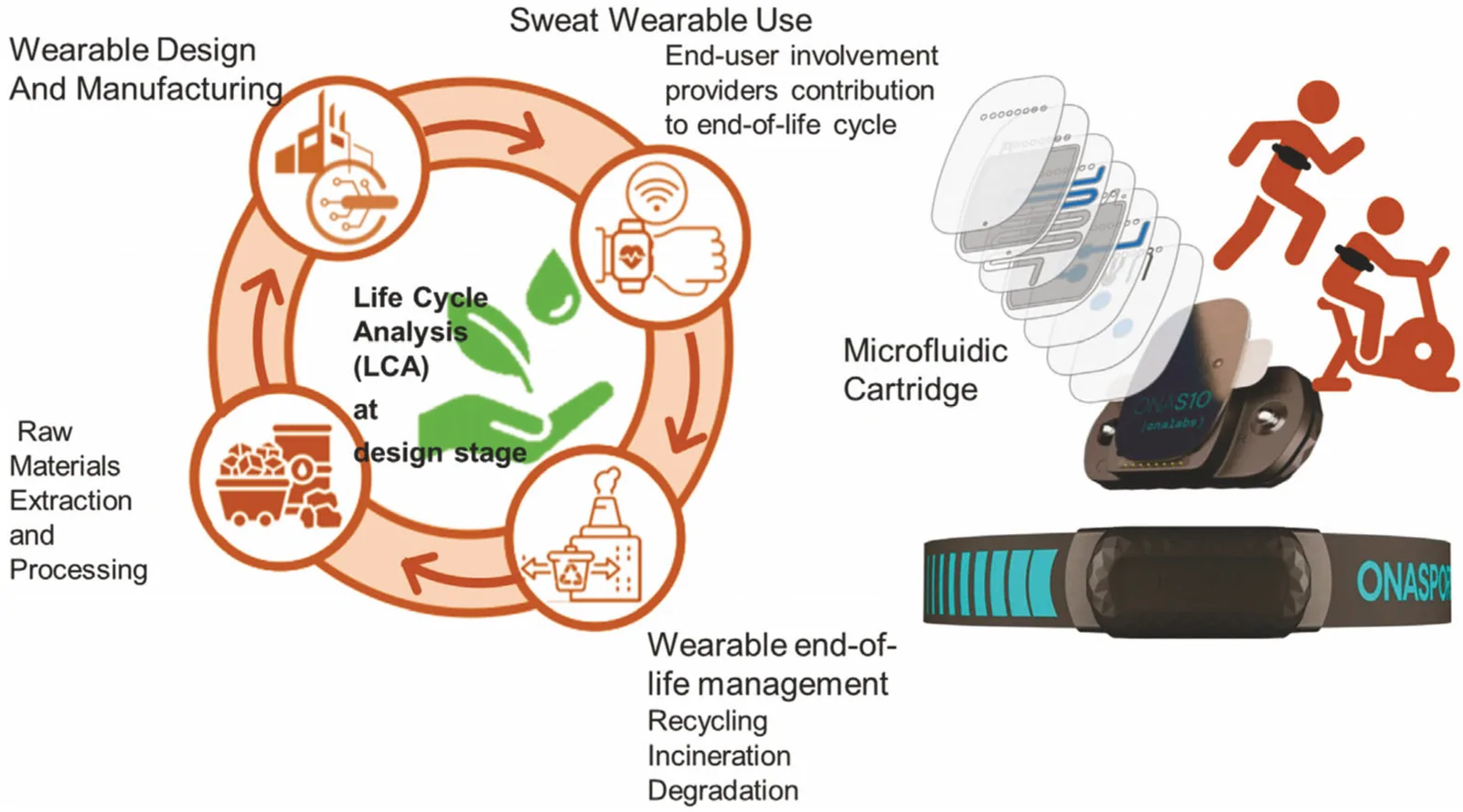
Life cycle assessment framework of a wearable sweat sensing system with emphasis on the microfluidic cartridge. The left panel illustrates the overall life cycle, encompassing stages from raw material extraction and processing through device fabrication, usage, and end-of-life management. The right panel presents the wearable system in operation, along with a detailed exploded view of the microfluidic cartridge highlighting its components for sweat collection, handling, and analyte detection [196].

The life cycle framework of the wearable sensor system is conceptually illustrated in Figure 19, which captures the interconnected stages from raw material extraction to end-of-life management. The figure presents a circular representation of the product life cycle, including material sourcing, device fabrication, user operation, and disposal or recycling pathways, thereby emphasizing the importance of evaluating environmental impact across the entire system rather than isolated stages. It further highlights the integration of life cycle assessment within the design process, enabling early identification of high-impact components and facilitating targeted improvements. The accompanying visualization of the wearable device and its microfluidic cartridge provides insight into how individual components contribute to both functionality and environmental burden. The inclusion of end-of-life scenarios such as recycling, incineration, and degradation underscores the importance of designing wearable sensors that support safe disposal and potential material recovery. Together, these results demonstrate that integrating life cycle assessment into the design of wearable photonic and electronic systems is essential for achieving sustainable, durable, and environmentally responsible technologies [196].

To provide a systematic perspective on the translation of self-powered wearable photonic sensors from laboratory-scale prototypes to scalable and sustainable technologies, it is essential to integrate considerations spanning fundamental physics, fabrication processes, material sustainability, and system-level performance. Table 4 summarizes these interdependent factors by linking governing physical mechanisms and process parameters with quantitative device metrics, scalability constraints, and environmental impact indicators, thereby offering a unified framework for evaluating manufacturability and real-world deployment.

## 9. Outlook: Toward Autonomous Wearable Photonic Systems

The continued evolution of self-powered wearable photonic sensors is expected to be driven by the convergence of advanced materials, intelligent data processing, and system-level integration strategies that extend beyond isolated sensing toward fully autonomous and adaptive platforms [214]. While significant progress has been achieved in demonstrating individual device concepts, the next stage of development will require coordinated advances that simultaneously address energy autonomy, mechanical resilience, signal reliability, and real-world usability [215]. This transition will depend not only on improving individual components but also on establishing unified design frameworks in which photonic sensing, energy harvesting, data processing, and user interaction are seamlessly integrated within a single wearable system [216,217].

A central enabler of such systems is the emergence of multifunctional materials capable of combining optical sensitivity, energy conversion, mechanical flexibility, and environmental stability within a single platform [218]. Advances in hybrid semiconductors, two-dimensional heterostructures, and nanocomposites have demonstrated the feasibility of integrating photovoltaic, thermoelectric, and piezoelectric functionalities in compliant formats, thereby reducing system complexity and enabling intrinsic coupling between sensing and energy generation [215,219]. The incorporation of self-healing polymers, stretchable conductors, and bioinspired materials further enhances durability and long-term reliability under continuous operation. Future efforts will likely focus on band structure engineering, defect control, and scalable fabrication techniques that preserve multifunctionality across large areas [159,220].

At the system-level, the integration of artificial intelligence offers a powerful approach to overcoming challenges associated with noisy signals, environmental variability, and user-dependent conditions [221]. Machine learning techniques can enable real-time signal denoising, feature extraction, and calibration, improving the accuracy of weak or fluctuating self-powered outputs. Beyond data processing, adaptive photonic systems may dynamically adjust their operation in response to environmental inputs or predicted energy availability, optimizing sensing modalities, duty cycles, and spectral response to balance energy efficiency with information fidelity [222]. The integration of low-power edge computing with photonic sensing platforms will be critical for enabling such adaptive functionality while maintaining energy autonomy [223].

In parallel, wearable photonic sensors are expected to evolve into components of closed-loop health monitoring systems, where continuous sensing is coupled with real-time analysis and responsive actuation [224,225,226]. Such systems could enable personalized interventions by linking optical monitoring of physiological parameters to feedback mechanisms such as drug delivery, thermal regulation, or electrical stimulation. The self-powered nature of these platforms is essential for ensuring uninterrupted operation in long-term healthcare applications. Achieving this vision will require seamless integration of sensing, actuation, energy management, and communication modules, supported by robust algorithms capable of interpreting multimodal data streams [227].

Despite these advances, several key challenges remain. The limited and variable energy available from ambient and body-derived sources constrains continuous operation and high-fidelity sensing, particularly under low illumination or fluctuating mechanical input [228]. Long-term material stability under combined mechanical, thermal, and chemical stress is not yet fully understood, especially for emerging low-dimensional and hybrid systems. In addition, the lack of standardized performance metrics and testing protocols hinders meaningful comparison across studies, while signal integrity issues such as motion artifacts [229], environmental noise, and device variability continue to affect accuracy and reproducibility [230]. Addressing these challenges will require interdisciplinary collaboration across materials science, device engineering, and data science [231].

The pathway toward commercialization will depend on advances in scalable manufacturing, cost reduction, and user-centered design. Techniques such as printing, roll-to-roll processing, and large-area deposition offer promising routes for producing flexible photonic devices, although maintaining uniform performance and reliability remains a challenge [232]. Integration with existing wearable platforms, including textiles and consumer electronics, will require compatibility with established manufacturing processes and regulatory standards, as well as careful consideration of data privacy, user comfort, and long-term safety [233]. A realistic development strategy involves the gradual incorporation of self-powered photonic components into hybrid systems, followed by progressive reduction in battery dependence as energy harvesting technologies mature. Ultimately, success in this field will depend on delivering robust, affordable, and user-friendly systems that provide tangible benefits in applications such as healthcare monitoring, fitness tracking, and environmental sensing [234].

## 10. Conclusions

Self-powered wearable photonic sensors represent a rapidly evolving class of technologies that bridge photonics, energy harvesting, and flexible electronics to enable energy-autonomous sensing platforms. This review has highlighted how diverse photophysical mechanisms, including photovoltaic, photogating, and triboelectric effects, can be harnessed to generate electrical signals directly from ambient or body-derived energy sources while simultaneously performing optical sensing. The integration of these mechanisms with advanced material systems such as hybrid semiconductors, two-dimensional heterostructures, and nanocomposites has significantly improved light absorption, carrier dynamics, and mechanical compliance, enabling seamless incorporation into wearable formats including textiles, flexible substrates, and epidermal devices.

At the device level, the evolution from conventional junction-based architectures to heterostructured and tandem configurations has enabled enhanced spectral selectivity, responsivity, and energy utilization. Co-design strategies that couple sensing and energy harvesting functions have emerged as a critical paradigm, particularly under realistic operating conditions where illumination intensity and spectral composition vary dynamically. Such approaches demonstrate that achieving true energy autonomy requires not only efficient energy conversion but also intelligent allocation of photonic resources between sensing and power generation.

Despite these advances, several fundamental and practical challenges remain. The limited and highly variable availability of ambient energy constrains continuous operation, especially in low-light indoor environments. Mechanical deformation, long-term material degradation, and environmental exposure can further impact device stability and performance. In addition, signal reliability remains a concern due to noise, motion artifacts, and variability in user conditions. These challenges highlight the need for improved materials with enhanced stability, device architectures that are tolerant to mechanical and optical fluctuations, and system-level strategies that incorporate adaptive operation and robust signal processing.

Looking forward, the convergence of multifunctional materials, adaptive photonic architectures, and intelligent data processing is expected to drive the next generation of autonomous wearable systems. The integration of low-power electronics and machine learning algorithms can enable real-time optimization of sensing performance and energy usage, while advances in scalable fabrication techniques will facilitate large-area and cost-effective production. Furthermore, the development of closed-loop systems that combine sensing, analysis, and actuation has the potential to transform wearable photonic devices from passive monitors into active components of personalized healthcare and human–machine interaction. In summary, self-powered wearable photonic sensors have demonstrated significant progress toward energy-autonomous operation, yet their widespread deployment will depend on overcoming challenges related to energy efficiency, stability, and system integration. Continued interdisciplinary efforts across materials science, device engineering, and data science will be essential to translate laboratory innovations into robust, user-friendly technologies. The insights presented in this review provide a foundation for guiding future research and accelerating the realization of practical, multifunctional wearable photonic systems.

## Figures and Tables

**Table 1:** Comparative overview of the principal photophysical mechanisms enabling self-powered operation in wearable photonic sensors.

Mechanism	Governing Principle	Bias Requirement	Spectral Response	Output Type	Representative Responsivity/Output	Typical Detectivity (Jones)	Response Time	Wearable Relevance	Key Limitations
Photovoltaic [37,54]	Built-in electric field at p–n junction, Schottky junction, or heterointerface separates photogenerated carriers	None (true zero-bias)	UV–Vis–NIR (material-dependent)	Current (A/W)	Responsivity: ~10^−3^–10^0^ A W^−1^	~10^10^–10^13^	μs–ms	Direct self-powered photodetection; compatible with thin, flexible devices	Limited internal gain; trade-off between flexibility, stability, and efficiency
Photothermoelectric [44,45]	Light absorption generates temperature gradient → Seebeck voltage	None	Broadband (UV–IR)	Voltage (V/W)	Voltage responsivity: ~10^−6^–10^−3^ V W^−1^	~10^6^–10^9^	ms–s	Effective under weak or broadband illumination; compatible with thermoelectric composites	Low signal magnitude; slow response due to thermal processes
Pyroelectric [48,54]	Time-varying temperature induces polarization change → transient current	None	Broadband (thermal/IR, modulated light)	Current (transient)	Effective responsivity: ~10^−4^–10^−2^ A W^−1^ (modulated signals)	~10^8^–10^10^	ms–s	Suitable for dynamic sensing in flexible ferroelectric materials	Cannot detect steady-state signals; depends on dT/dt
Piezo-phototronic [47,49,50]	Strain-induced piezopotential modulates carrier separation and transport	None	UV–Vis (typically)	Current (A/W)	Responsivity: ~10^−3^–10^0^ A W^−1^	~10^9^–10^12^	μs–ms	Enables coupling between mechanical deformation and photoresponse in wearables	Requires controlled strain; sensitive to interface quality
Triboelectric hybrid [51]	Contact electrification + electrostatic induction; light modulates charge transfer	None	UV–Vis (device-dependent)	Voltage/Charge (nonlinear)	Output waveform-dependent (not standardized)	Not standardized	ms–s	Suitable for motion-rich wearable environments; combines sensing and energy harvesting	Nonlinear output; humidity sensitivity; poor standardization
Photogating (self-/near-zero bias) [55,56]	Trapped photocarriers modulate channel conductance → internal gain	Near-zero bias (rarely true zero-bias)	UV–NIR	Current (A/W, gain-enhanced)	Responsivity: ~10^−2^–10^1^ A W^−1^	~10^10^–10^13^	ms–s	High sensitivity for weak-light detection	Slow recovery; hysteresis; instability due to trap states

The listed values are representative literature ranges under different test conditions; the references given in each row identify the relevant source studies. Detectivity values are reported as provided by the original authors and may be based either on dark current shot-noise calculations or measured noise spectra. Direct comparison requires the same wavelength, optical power, bias, bandwidth, and noise method [57].

**Table 2:** Advanced classification of device architectures for self-powered wearable photonic sensors.

Architecture Class	Representative Device Structures	Underlying Transduction Mechanism	Carrier Generation & Transport Physics	Bias Requirement/Energy Autonomy	Representative Performance Metrics	Key Advantages	Primary Limitations/ Failure Mechanisms	Wearable Integration Considerations
Photovoltaic Photodetectors [54,83]	p–n junctions, Schottky junctions, van der Waals heterostructures	Photovoltaic effect	Photon absorption → exciton generation → drift-driven separation in built-in electric field; recombination governed by Shockley–Read–Hall (SRH) and interface states	True self-powered (zero bias)	Responsivity: ~10^−3^–10^0^ A W^−1^; detectivity: ~10^10^–10^13^ Jones; response time: μs–ms	Fast response; low noise; direct signal generation; simple readout circuitry	Limited internal gain; recombination losses; interface defects; band alignment sensitivity	Requires flexible transparent electrodes (e.g., AgNW, graphene); stable junctions under strain; moisture/oxygen encapsulation
Photogating-Based Architectures [55,89]	Graphene–quantum dot hybrids, MoS_2_ phototransistors, hybrid 2D heterostructures	Photogating (trap-mediated gain)	Photoexcited carriers trapped in sensitizer layer induce electrostatic gating; channel transport dominated by high-mobility conduction (e.g., graphene)	Near-zero bias; true self-powered operation uncommon	Responsivity: ~10^−2^–10^5^ A W^−1^ (device-dependent); detectivity: up to ~10^14^ Jones; response time: ms–s	Extremely high sensitivity under low illumination; internal gain; broadband detection	Slow response due to trap dynamics; hysteresis; drift; environmental sensitivity (humidity, temperature)	Requires controlled trap density; environmental passivation; mechanical deformation affects trapping kinetics and stability
Waveguide-Integrated Photonic Sensors [70,106]	Polymer core–cladding waveguides, elastomeric optical networks with embedded µLEDs and photodetectors	Optical intensity modulation via optomechanical coupling	Light propagation via total internal reflection; evanescent field interaction; mechanical perturbations alter mode confinement and transmission	Externally powered or hybrid (not intrinsically self-powered)	Optical loss: device-dependent (<1–few dB cm^−1^); spatial resolution: sub-mm; response time: ms	Distributed and multiplexed sensing; decoupling of sensing and detection regions; high spatial resolution	Optical loss under bending; mode leakage; coupling inefficiencies; alignment sensitivity	Requires refractive index stability under strain; robust optical coupling; integration with flexible light sources and detectors
Fiber-Based and Textile-Integrated Sensors [86,100,107,108]	Optical fibers, fiber Bragg gratings (FBGs), polymer waveguides embedded in textiles	Intensity, wavelength, or phase modulation (e.g., microbending loss, Bragg shift)	Light propagation modulated by strain, pressure, or environmental interaction, scattering and absorption in deformable media	Typically, hybrid (can integrate energy harvesting modules)	Strain sensitivity: pm/με (FBG); large-area distributed sensing; response: ms–s	Seamless textile integration; large-area coverage; low transmission loss over distance	Mechanical fatigue; microcracking; washing-induced degradation; signal drift from deformation	Requires protective coatings; washability; stable fiber–textile interfaces; mechanical reinforcement
Ultrathin/Imperceptible Photonic Devices [109,110]	Ultrathin OPVs, OLED–OPD integrated systems, transparent electrodes, nanomembrane devices	Primarily photovoltaic + photodetection coupling	Carrier generation and transport in ultrathin films; interface-limited transport; reduced strain via neutral mechanical plane design	Self-powered or hybrid (often PV-integrated)	Thickness: <1–5 µm; optical transmittance: >70%; response: ms (device-dependent)	Conformal skin integration; minimal motion artifacts; improved optical coupling; user comfort	Reduced optical absorption due to thin active layers; electrode degradation; mechanical fragility	Requires ultrathin encapsulation; strain-neutral mechanical design; biocompatibility; stable skin adhesion

The listed values are representative literature ranges under different test conditions; the references given in each row identify the relevant source studies.

**Table 3:** Representative co-design strategies for integrating photonic sensing and energy harvesting in self-powered wearable systems.

Co-Design Strategy	Operating Concept	Benefits	Limitations	Best-Suited Photonic Mechanisms	Wearable Relevance
Direct self-powered transduction [134]	The sensing stimulus directly generates an electrical output without external bias	Minimal circuit complexity; true zero-bias sensing	Limited freedom to optimize sensing and power independently	Photovoltaic, photothermoelectric, pyroelectric [54]	Highly attractive for thin, lightweight skin-mounted sensors
Shared optical pathway for sensing and harvesting [83,135]	Incident light contributes simultaneously to sensing and partial energy supply	Efficient use of available photon flux	Trade-off between spectral selectivity and energy conversion	Photovoltaic, photogating	Useful for ambient light wearable sensing
Spectrally decoupled co-design [136,137]	Different spectral bands are allocated to sensing and energy harvesting	Better functional optimization	Requires optical filtering or multilayer design	Photovoltaic + wavelength-selective architectures	Valuable in PPG, pulse oximetry, and multispectral sensing
Multisource hybrid harvesting [138,139]	Optical sensing is combined with solar, thermal, or mechanical energy harvesting	More robust operation in variable environments	Higher integration and power management complexity	PV + triboelectric + thermoelectric hybrids	Important for continuous wearable use under changing conditions
Integrated storage-assisted operation [140,141,142]	Harvested energy is buffered in microsupercapacitors or related storage units to support sensing continuity	Stabilizes intermittent power input	Adds footprint and packaging complexity	Applicable to all mechanisms at system level	Useful for duty-cycled and low-light wearable systems
Adaptive energy-aware system design [143,144]	Sampling, processing, and transmission are adjusted according to available harvested energy	Improves autonomy and data fidelity	Requires ultra-low-power control electronics	Applicable to all mechanisms at system level	Important for real-world autonomous wearables

**Table 4:** Physically grounded framework for scalable manufacturing, sustainability, and system-level deployment of self-powered wearable photonic sensors.

Domain	Sub-Domain	Governing Physics/Mechanisms	Critical Process Parameters	Device-Level Metrics (Quantitative)	Scalability Bottlenecks	Sustainability & Environmental Metrics	System-Level Implications
Manufacturing Platforms	Roll-to-roll (R2R) coating [180,183,184]	Thin-film fluid dynamics governed by Capillary number (Ca = μU/γ), Péclet number (Pe), solvent evaporation kinetics, and Marangoni flow	Web speed (0.1–10 m·min^−1^), coating thickness (10 nm–10 µm), drying temperature (25–150 °C), ink viscosity (1–1000 mPa·s)	Thickness uniformity (<±5%), sheet resistance (10–10^3^ Ω/sq), defect density (<10^2^–10^4^ cm^−2^), yield (70–99%)	Nonuniform drying, edge instabilities, multilayer misalignment, solvent incompatibility	Solvent usage (g·m^−2^), energy consumption (kWh·m^−2^), material utilization (>80%)	Enables continuous large-area fabrication; critical for textile-scale and epidermal devices
	Printing (inkjet, gravure, aerosol jet) [181,182,183,200]	Droplet formation governed by Reynolds (Re), Weber (We), and Ohnesorge (Oh) numbers; capillary flow and coffee-ring effect	Drop spacing (10–100 µm), nozzle diameter (1–50 µm), substrate surface energy (20–60 mN·m^−1^)	Resolution (10–50 µm), roughness (<10–100 nm RMS), electrical variability (<5–20%)	Ink instability, nozzle clogging, poor layer registration, limited multilayer stacking precision	Additive material efficiency (>90%), solvent toxicity (VOC emissions), waste reduction	Enables heterogeneous integration of active layers, electrodes, and encapsulation
Materials Systems	Semiconductors (organic, perovskite, 2D) [201,202]	Carrier generation and recombination (Shockley–Read–Hall statistics), exciton diffusion, ion migration (perovskites), bandgap engineering	Stoichiometry, crystallization rate, defect density, doping concentration	Mobility (10^−3^–10^2^ cm^2^·V^−1^·s^−1^), carrier lifetime (ns–ms), detectivity	Instability (moisture, oxygen), ion migration, large-area uniformity limitations	Toxicity (e.g., Pb content < 0.1 wt%), recyclability, elemental abundance	Determines sensing efficiency, stability, and regulatory acceptance
	Electrodes (AgNW, graphene, CNT)	Electrical percolation theory; contact resistance; plasmonic losses	Network density, junction resistance, thickness (10–500 nm)	Sheet resistance (10–100 Ω/sq), transmittance (>80–95%)	Trade-off between transparency and conductivity; degradation under strain	Use of scarce metals (Ag, Au); carbon-based alternatives improve sustainability	Affects optical coupling, flexibility, and device lifetime
	Substrates & encapsulants [203,204]	Diffusion governed by Fick’s law; mechanical strain distribution; modulus mismatch	Young’s modulus (kPa–GPa), thickness (1–100 µm), permeability	Water vapor transmission rate (WVTR: 10^−6^–10^−3^ g·m^−2^·day^−1^), bend radius (<1–10 mm)	Barrier–flexibility trade-off; delamination under cyclic strain	Biodegradability, recyclability, polymer aging	Controls durability, comfort, and skin conformity
Encapsulation & Packaging [205]	Barrier layers	Multilayer diffusion barriers; tortuosity effects; interface adhesion	Layer thickness (10 nm–µm), number of layers, interface quality	WVTR (<10^−6^ g·m^−2^·day^−1^ for high stability), lifetime (10^3^–10^5^ h)	Pinholes, cracking under strain, optical absorption losses	Reduced degradation → extended device lifetime	Essential for stability of organic and perovskite devices
	Mechanical protection	Fracture mechanics; fatigue (Paris’ law); neutral mechanical plane design	Strain tolerance (1–50%), cyclic loading (>10^4^–10^6^ cycles)	Crack density, resistance change (<5–10% after cycling)	Fatigue-induced failure, interconnect fracture	Increased lifetime reduces environmental footprint	Enables washability and long-term wearable operation
Process Integration [206,207]	Multilayer device stacking	Interface recombination (Shockley–Read–Hall), band alignment, interdiffusion kinetics	Annealing temperature (≤150–300 °C for flexible substrates), layer sequence	Interface defect density, recombination rate, device yield (70–95%)	Incompatible solvents/thermal budgets; layer damage	Higher process complexity → higher energy consumption	Determines efficiency, reproducibility, and manufacturability
Regulatory & Clinical Translation	Standardization & testing [208]	Variability in optical coupling, skin scattering, and illumination conditions	Calibration protocols, reference illumination (lux), test duration	Accuracy (<±5%), reproducibility, inter-device variation (<10%)	Lack of standardized benchmarks for self-powered devices	Enables reliable comparison and reduces waste from failed designs	Critical for commercialization and clinical adoption
	Biocompatibility & safety [209]	Skin–device interface chemistry; ion diffusion; thermal and electrical safety	Cytotoxicity testing (ISO 10993), temperature rise (<2–5 °C)	Cell viability (>70–90%), irritation response, failure rate	Toxic materials (Pb, solvents), long-term exposure risks	Drives shift toward non-toxic, skin-safe materials	Required for medical-grade wearable deployment
System-Level Scalability	Energy–sensing integration [210]	Coupled optoelectronic conversion; power balance; load matching	Power density (µW–mW·cm^−2^), responsivity (10^−3^–10^1^ A/W)	Energy autonomy ratio (>1 for self-powered), duty cycle	Mismatch between harvested power and consumption	Efficient systems reduce energy waste	Enables fully autonomous wearable platforms
	Manufacturing yield & cost [211,212,213]	Statistical process variation; defect tolerance	Yield (70–99%), cost ($10–$1000 m^−2^ depending on technology)	Cost–performance ratio	Yield loss in multilayer systems	Higher yield reduces waste and cost	Determines commercial feasibility

The numerical values in this table are indicative engineering and manufacturing ranges, rather than directly comparable photodetector-performance records.

## Data Availability

No new data were created or analyzed in this study.
